# Patient-reported outcome and experience measures in cardiovascular disease: a scoping review as part of iCARE4CVD

**DOI:** 10.1186/s41687-025-00980-4

**Published:** 2025-12-09

**Authors:** Bianca Steiner, Anne Neumann, Markus Schwertfeger, Marlo Verket, Iñaki Romero, Carmen Hurtado, Vianne Schiefel, Anne McNulty, Sathish Sankarpandi, Arno J. Gingele, Thomas M. Helms, Hans-Peter Brunner-La Rocca, Bettina Zippel-Schultz

**Affiliations:** 1 German Foundation for the Chronically Ill, Pariser Platz 6, 10117 Berlin, Germany; 2 Faculty of Healthcare, Ostfalia University of Applied Sciences, Poststr. 19, 38446 Wolfsburg, Germany; 3 Roche Diagnostics International Ltd, Forrenstrasse 2, Rotkreuz, 6343 Switzerland; 4 Department of Internal Medicine I, University Hospital Aachen, RWTH Aachen University, Pauwelsstrasse 30, 52074 Aachen, Germany; 5 Huawei Technologies R&D Europe, Philipssite 5, Leuven, 3001 Belgium; 6 Breakthrough T1D, 200 Vesey Street, New York, NY 10281 USA; 7 School of Nursing and Midwifery, Queen’s University Belfast, 97 Lisburn Road, Belfast, BT9 7BL UK; 8 VirtTuri Ltd, Cedar House Creeting Road East, Stowmarket, IP14 5BT UK; 9 Cardiology Department, Maastricht University Medical Centre+, P. Debeyeplein 25, Maastricht, The Netherlands; 10 Cardiovascular Research Institute Maastricht (CARIM), Maastricht University, Universiteitssingel 50, Maastricht, The Netherlands

**Keywords:** Cardiovascular diseases, Patient reported outcome measures, Health status, Quality of life, Patient-reported experience measures, Quality of health care, Digital health, Scoping review

## Abstract

**Background:**

Patient-reported outcome and experience measures (PROMs and PREMs) are increasingly acknowledged as vital instruments for assessing the quality of care for cardiovascular diseases (CVD). These measures include validated and non-validated questionnaires, interview guides, and workshops, which differ in terms of their structure, reliability, and application. Currently, there is no overview of which patient-reported outcomes and experiences are measured in CVD research and care, and there is limited consensus on how PROMs and PREMs are selected and applied.

**Methods:**

A scoping review was conducted in accordance with the PRISMA extension for scoping reviews, with the aim of systematically identifying and analysing studies that report on the use of PROMs and PREMs in CVD. Literature searches were performed in PubMed and ClinicalTrials.gov for studies published before April 2024. Studies assessing patients with heart diseases (ICD-10: I20–I25; I34–I37; I42; I46–I49; I50) using conventional or digital measures to evaluate care quality from the patient’s perspective were included. Studies focusing on CVDs stemming from neurological complications, rheumatic disease, birth defects, and peripheral artery disease were excluded. The same applies to studies using non-validated PROMs. Data analysis was conducted using qualitative content analysis.

**Results:**

Of the 5,489 records identified, 390 publications were included for full-text analysis. More than a third of these were observational studies (*n* = 168; 43%). PROMs were used more frequently than PREMs (309 vs. 159 studies). Quality of life was the most measured patient-reported outcome (211 of 309 studies; 68%), followed by health status (*n* = 110; 36%) and depression (*n* = 105; 34%). A total of 540 instances of PROM application were recorded across the 390 identified studies, representing 140 unique measurement instruments. More than half of these are disease-specific (*n* = 443; 57%), particularly for heart failure, the condition most frequently studied. Of the 140 different PROMs identified, 26 were used in more than five studies, indicating that while many tools exist, only a small subset are in common use. Of the 166 PREMs identified, 57% (*n* = 94) were self-developed questionnaires or interview guides, predominantly used in qualitative studies. Validated PREMs often do not focus on a specific disease. The most frequently assessed patient-reported experiences were self-care, treatment experiences, satisfaction, knowledge, and adherence. Both patient-reported outcomes and experiences were primarily assessed using paper-based measures.

**Conclusion:**

This review highlights the lack of consensus in the use, validation status and reporting of PROMs and PREMs in CVD research and care. Although validated, disease-specific PROMs are widely used for key outcomes such as quality of life, there is a lack of standardised PREMs tailored to CVDs. To advance patient-centred care in CVDs, there is a need for more consistent use of validated instruments, transparent reporting of administration methods, and the development of robust PREMs. Furthermore, standardising the PROMs and PREMs used may help, particularly with regard to the comparability of findings in different studies.

**Supplementary Information:**

The online version contains supplementary material available at 10.1186/s41687-025-00980-4.

## Introduction

### Background

Cardiovascular disease (CVD) is one of the leading causes of death in Europe and worldwide, with an immense burden on society [1]. CVD has a great impact on the lives and wellbeing of those affected, as well as their relatives. The combination of an ageing population, unhealthy lifestyle, and increased numbers of risk factors has led to a rise in the number of patients with CVD [2]. CVD is a multifactorial disease with individual risk factors such as a positive family history, genetic background, hypertension, diabetes (type 1, or type 2), obesity, dyslipidaemia, smoking, and chronic kidney disease [2]. CVDs progress through various stages [3]:

1. (Early) risk to develop CVD, irrespectively if treated or not, but absence of any of the following 3 stages.
2. Established CVD with normal cardiac function defined as the absence of structural or functional cardiac abnormalities, e.g. on echocardiography.
3. Structural and/or functional cardiac abnormality without the diagnosis of heart failure (HF) – i.e. no (previous) signs or symptoms of HF (as defined by the 2021 Heart Failure guidelines of the European Society of Cardiology) [4].
4. Chronic HF (independently of left ventricular ejection fraction), i.e. structural and/or functional cardiac abnormalities and (previous) signs and/or symptoms of HF.

Timely assessment of risk is needed for early diagnosis and intervention if required. Personalizing prevention and treatment of CVD will optimize healthcare for patients. However, to do so successfully, a deeper understanding of the needs of patients and their relatives is pivotal. Moreover, in order to assess quality of care (processes), it is not enough to consider classic quality indicators, such as patient safety, timelines of care, effectiveness and appropriateness, but also patients` personal perceptions. Patients play a crucial role in assessing whether a care process aligns with their preferences and wishes [5]. Therefore, patient-reported outcome measures (PROMs) and patient-reported experience measures (PREMs) are becoming increasingly important as patient-centred instruments for obtaining a holistic view on health care quality and orienting healthcare provision towards patients and their needs [5]. Patients cannot only provide details regarding their outcomes, but also express and assess their general condition and the treatment process as a whole. PROMs, on the one hand, are used to capture patients` perceptions of their health and disease, thereby providing immediate feedback on individual care as well as insights into longer-term clinical outcomes [6, 7]. For this purpose, indicators such as symptoms, distress, quality of life (QoL), functional ability or self-reported health status are measured. PREMs, on the other hand, assess patients` experiences with healthcare delivery and care processes. They capture constructs that are crucial to the quality of individual care, such as communication, involvement in decision-making processes, waiting times, and patients’ understanding of their care pathways [7, 8].

PROs and PREs are measured by standardized or non-standardized questionnaires. These might be self-designed or adapted tools. Examples of standardized instruments include the EQ-5D of EuroQol and the Short Form-36 Health Survey (SF-36), while non-standardized, project-specific surveys are developed to capture particular aspects of patient experience or outcomes. Studies indicate that the use of patient-reported outcomes (PROs) and patient-reported experiences (PREs) may improve outcomes and prevention by enabling early detection of worsening symptoms and providing personalised, appropriate care tailored to patients’ needs [9–11].

Although previous studies have demonstrated the growing use of PROMs and PREMs in evaluating care for chronic diseases, the available evidence for CVD remains fragmented. Existing reviews often focus exclusively on PROMs [12, 13], single diseases, such as heart failure [14, 15], or specific interventions [16, 17]. However, no comprehensive synthesis jointly examines PROMs and PREMs across the CVD spectrum. In particular, it is unclear, whether there are differences in relation to the different stages of CVD and thus patients` different expectations and requirements. This urgent need for in-depth knowledge is to be addressed as a component of the iCARE4CVD project that aims to develop a nuanced understanding of CVD [3] (Innovative Health Initiative Joint Undertaking, grant agreement no. 101112022.).

### iCARE4CVD

Individualised care from early risk of cardiovascular disease to established heart failure (iCARE4CVD) is a European research initiative involving academic and industry partners. The project aims to improve the prevention and treatment of CVD by providing personalised, data-driven healthcare [3]. Over the course of four and a half years, the project will seek to enhance current care by enabling the earlier detection of cardiovascular risk, refining patient stratification to identify those in need of more intensive intervention and developing artificial intelligence-based tools to predict individual treatment responses. A key aspect of the project is integrating patient perspectives to ensure that patient-relevant outcomes are embedded in its development and evaluation.

### Objectives

This scoping review aimed to identify PROMs and PREMs used to assess the patients’ perspectives on the quality of care provided in the treatment of CVDs, with a particular focus on heart diseases. Thereby, quality of care is not used as a single theoretical construct, but as an overarching concept that captures patient-reported outcomes and experiences related to the process and its results. This review provides an overview of contexts in which PROMs and PREMs are applied, including the characteristics of the population and the nature of interventions, the specific outcomes assessed, the dimensions of PREs measured, and the instruments employed. The analysis is structured around three key categories: (A) Setting, (B) PROMs, and (C) PREMs. This review seeked to answer the following questions:

A. Setting
  1. Which CVDs are the measures used for?
  2. Which CVD-relevant comorbidities do the patients have?
  3. What interventions/health services do the patients receive?
  4. For what purpose are the measures used?
B. PROMs
  1. Which PROs are measured?
  2. Which validated measurement instruments are used?
  3. What format is used for the survey?
C. PREMs
  1. Which PREs are measured?
  2. Which measurement instruments are used?
    If non-validated questionnaires are used, the following questions need to be answered additionally:
    i. Which dimensions are covered to assess these PREs?
    ii. Which individual aspects/items are asked for?
    iii. Which quality criteria for evidence (validity, reliability, and objectivity) does the measuring instrument fulfil?
  3. What format is used for the survey?

## Methods

The review protocol for this literature study is based on the extended PRISMA checklist for scoping reviews PRISMA-ScR [18]. Given the exploratory nature of the research question, a scoping review appeared to be a more appropriate and consequently a more effective approach than a systematic review to gain insights into the use of PROMs and PREMs to measure care quality in CVD and derive relevant hypotheses.

The study is registered with OSF (registration DOI: 10.17605/OSF.IO/BSXNT).

### Eligibility criteria

In **general**, peer-reviewed articles, reviews, and conference publications published before April 2024 were included (Table 1). Articles published in English, German, Dutch, French, and Italian were considered.

In terms of the **population**, all publications focusing on patients with CVD were included (Table 1). In accordance with the International Classification of Diseases 10th Revision (ICD-10), this includes the following diseases: coronary artery disease (CAD), acute or chronic HF, cardiac arrythmias, angina pectoris due to CAD, myocardial infarction due to CAD, and chronic ischemic heart disease. CVDs stemming from neurological complications, rheumatic disease, birth defects, and peripheral artery disease were excluded due to the difference in their management and treatment strategies. Furthermore, patients in stage 1 (individuals at an early risk of developing CVD) were excluded. Including this group would have meant considering a very large number of studies focusing on isolated risk factors rather than established CVD. This decision ensured a clear focus on studies involving patients with evident CVD.

In terms of the **outcome**, all publications using conventional or digital measures to assess the quality of care from patients` perspective were included. Thereby, PREMs used as performance indicators at the level of healthcare systems, e.g., for evaluating national healthcare system strategies (*macro-level*), and healthcare providers, for example for performance evaluation and quality-based financing (*meso-level*) were excluded [19]. This means that only PREMs used for improving the patients experience itself were considered (*micro-level*). As there are few validated PREMs in CVD research and care, studies using non-validated measurement instruments were also included [8]. This was done to avoid overlooking relevant evidence and to establish a more comprehensive basis for mapping the current research landscape. In contrast, for PROMs, only studies using validated questionnaires were included. This was because PROMs are significantly more widespread than PREMs, resulting in a much larger number of available instruments. Including only validated questionnaires ensures that the instruments used have undergone a systematic assessment of their psychometric properties, such as reliability, validity and responsiveness.

**Table 1:** Inclusion and exclusion criteria

Category	Inclusion criteria	Exclusion criteria
Type of publication	1. Peer-reviewed article, peer-reviewed conference article, reviews	1. Abstracts, poster, case reports, letters, editorial comment, position paper, brief reports, study protocols, validation studies on already validated questionnaires
	2. Publication before April 2024	2. Article not in English, German, Dutch, French, Spanish, or Italian
Population	3. Focus on patients with CVDs in stage 2 to 4 a. Ischaemic Heart Diseases (I20-I25) b. Non-rheumatic valvular heart disease (I34-I37) c. Cardiomyopathy (I42) d. Cardiac arrythmias (I46-I49) e. Heart failure (I50)	3. Main focus on CVDs stemming from neurological complications, rheumatic disease, birth defects, and peripheral artery disease (Q20-Q28; I05-I09; I63.9; I65.2; I67.2; I67.9; I70; I71; I73) 4. Focus on CVDs in stage 1 (risk factors) 5. Focus on other diseases (not CVD)
Outcome	4. Conventional or digital measures are used to assess the quality of care from patients` perspective	6. No measurement of PRE or PRO 7. PREMs: Measurement of PRE at the macro- and meso-level 8. PROMs: Non-validated measurement instrument used for PRO measurement

### Information sources

To gain a comprehensive overview, the biomedical and life sciences literature databases Medline and PubMed Central (PMC) were searched via PubMed. This was supplemented by a search of ClinicialTrials.gov.

### Search strategy

The search string was defined iteratively based on the PICO framework [20]. According to the objectives of this review, the key elements ‘patient, population, problem’ and ‘outcome’ are particularly relevant. The aspect ‘**patient**,** population**’ was described by using various terms for *cardiovascular disease* and appropriate synonyms. A conscious decision was made not to search explicitly for specific CVDs to avoid pre-selection (*bias*). Nevertheless, it was assumed that different CVDs are mapped using the associated MeSH-term ‘Cardiovascular Disease’.

The key element ‘**outcome**’ contains numerous PROs yet known and yet unknown. Therefore, various terms for PROs, PREs, PROMs and PREMs as well as appropriate synonyms and abbreviations were used for the search. Again, a conscious decision was made not to search for already known PROs, such as QoL, pain, distress, waiting time, or satisfaction, to avoid a pre-selection and thus a bias. As it was assumed that the concepts PROMs and PREMs are not necessarily mentioned explicitly, the search block also included an unspecific search for *patient perspective*, *patient journey* and *health care surveys* defined as “*statistical measures of utilization and other aspects of the provision of health care services including hospitalization and ambulatory care*” [21]. See Appendix 1 for the full search string.

ClinicalTrials.gov was queried in accordance with the pre-defined search string using the “Condition/disease” field for searching keywords related to CVDs and the “Other terms” field for keywords related to PROMs and PREMs (see Appendix 1). No date or other filters were applied.

### Synthesis of results

The summarization, analysis and evaluation of the full texts classified as relevant was based on the qualitative content analysis according to Mayring [22]. This basic procedure was adapted using Schreier’s toolbox for content-structuring qualitative content analyses [23]. The qualitative data analysis software MAXQDA (Version 24.9.1) was used to simplify and partially automate the analysis. Table 2 describes the main steps and approaches used.

**Table 2:** Steps of the qualitative content analyses (according to [23])

Main steps	Used approach
*Creating the category system*	
Basic strategy	Deductive-inductive
Deductive categorization	Setting – CVD, Stage of disease, Comorbidity, Intervention, Purpose PROM – PRO, Dimension, Item PREM – PRE, Dimension, Item Measurement Instruments – Measurement Instrument, Evidence, Limitations
Inductive categorization	Subsumption
Persons involved	2 (medical informatician, health scientists)
Termination criterion	None
*Division of material*	
Unit	Coding unit
Coding unit	Individual words and phrases
Systematic	Marking and coding in one step
Persons involved	1
*Coding*	
Test coding	Yes
Person involved in main coding	3

Information on PROMs and PREMs from ClinicalTrial.gov was added as far as possible in a last step. Besides title, acronym and the start date, the following study characteristics were extracted: (1) status; (2) results available; (3) conditions; (4) interventions; (5) outcome measures; (6) gender; (7) age; (8) study phase; (9) enrolment; (10) study type; (11) study design.

### Selection, categorization, and data extraction

The search request was made to PubMed on April 04, 2024. Title and abstract screening were conducted by 7 reviewers (AM, BS, BZS, IR, MS, MV, SS) with different background – medical informatics, business management in healthcare, public health, and engineering. Checking of predefined exclusion criteria was done for each publication sequentially by two reviewers. Whenever disagreements arose, a third reviewer (AN) with expertise in health psychology and education was consulted for decision-making. Full-text screening was carried out in the same way as the title and abstract screening. Following full-text screening, it was decided that reviews should be excluded from the analysis to avoid duplicating data and the risk of double-counting primary studies. This approach ensured that the analysis included only original research with primary data, allowing for a more precise and unbiased synthesis of findings. Qualitative content analysis was performed by three reviewers (AN, BS, VS).

## Results

**Fig. 1:**
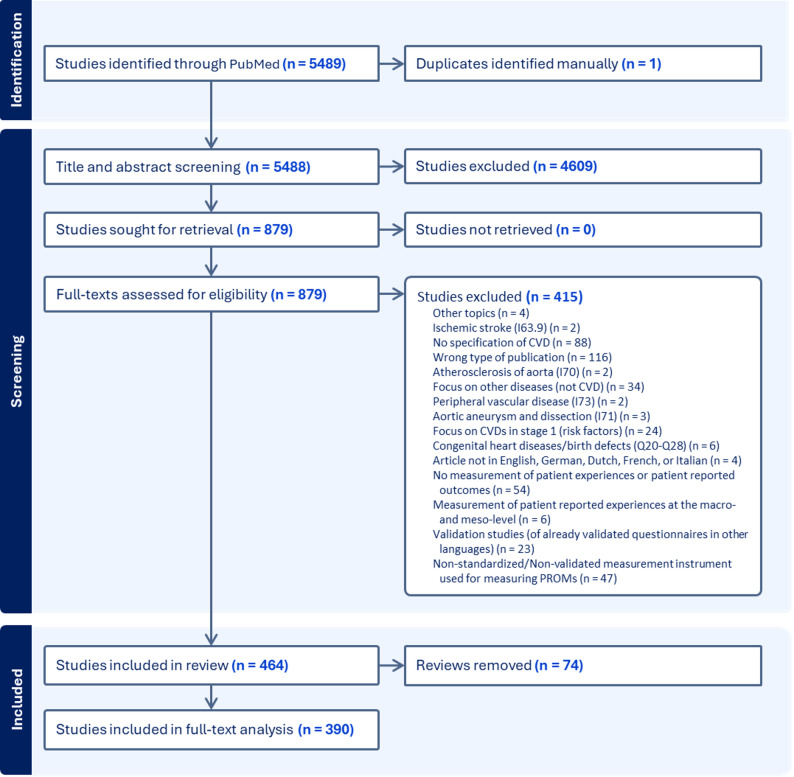
PRISMA flow diagram

The literature search identified 5489 publications (Fig. 1). After removing duplicates, 5488 publications remained for title and abstract screening. Of these, 879 were included in the full-text screening. Consequently, a total of 390 articles, published between 1998 and 2024, were selected for full-text analysis (see Appendix 2).

The search request was made to ClinicalTrials.gov on May 22, 2024. The search returned 758 matches. ClinicalTrials.gov study records (title and summary) were individually reviewed by one reviewer (CH) to identify and collect specific PROMs and PREMs. In total, 185 trials were included for analysis.

### General characteristics of included studies

In the 390 studies analyzed, PROs were collected nearly twice as often as PREs to assess patients’ perspectives in CVD care (Table 3). Of all studies using PROMs, more than half (*n* = 178; 58%) measured multiple PROs, e.g. fatigue and QoL. With PRE, this applied to fewer than a third of studies (*n* = 45; 28%). Typically, patients’ perspectives were assessed exclusively through PROMs, without incorporating PREMs (*n* = 231; 59%). A total of 81 studies (21%) relied solely on PREMs, while one fifth of studies (*n* = 78; 20%) recorded both PROs and PREs.

**Table 3:** Number of studies included grouped by study type

Study type	Count of studies (*n* = 390)	Studies measuring PROs (*n* = 309)	Studies measuring PREs (*n* = 159)
Feasibility study	7	7	3
Mixed-methods-study	5	4	3
Non-randomized clinical trial	6	5	3
Non-randomized study	8	8	1
Not defined	11	9	5
Observational study	168	144	61
Pilot study	11	10	7
Psychometric evaluation	19	17	5
Qualitative study	23	2	22
Quantitative study	43	22	26
Randomized controlled trial	73	68	19
Register study	16	13	4

### Setting

#### Study type and purpose

More than a third of the studies included in the full text analysis were observational studies (*n* = 168; 43%). Thereby, cohort studies (*n* = 77; 46%) and cross-sectional studies (*n* = 61; 36%) were carried out most frequently (Table 3). Randomized controlled trials (RCTs; *n* = 73; 19%), and quantitative studies (*n* = 43; 11%) followed in second, and third place. In RCTs and register studies, the proportion of PROs measured was substantially higher compared to PREs (54:14; 12:1). For feasibility studies, mixed-methods-studies and non-randomized clinical trials, the proportion was quite similar. PREs were most commonly measured in observational studies (*n* = 61; 38%), quantitative studies (*n* = 26; 16%), and qualitative studies (*n* = 22; 14%). Notably, in qualitative studies, PREs were recorded significantly more often than PROs.

Most studies measuring patient outcomes aimed to derive associations between outcomes, patient characteristics, and other factors (*n* = 115; 29%). The most frequently analysed associations were those between PROs and clinical outcomes (*n* = 13; 17%), PROs and demographics (*n* = 15; 13%), and between different PROs (*n* = 13; 11%). The second most common purpose of the studies was to evaluate therapies and healthcare services (*n* = 105; 27%). Usual care (*n* = 29; 25%), invasive therapies such as Transcatheter Aortic Valve Implantation (*n* = 23; 20%) and drug therapies (*n* = 21; 20%) were mostly evaluated for their effects or benefits. Studies simply focusing on the assessment of the patient’s perspective in general measured PREs significantly more frequently than PROs (50:21).

#### Population

**Fig. 2:**
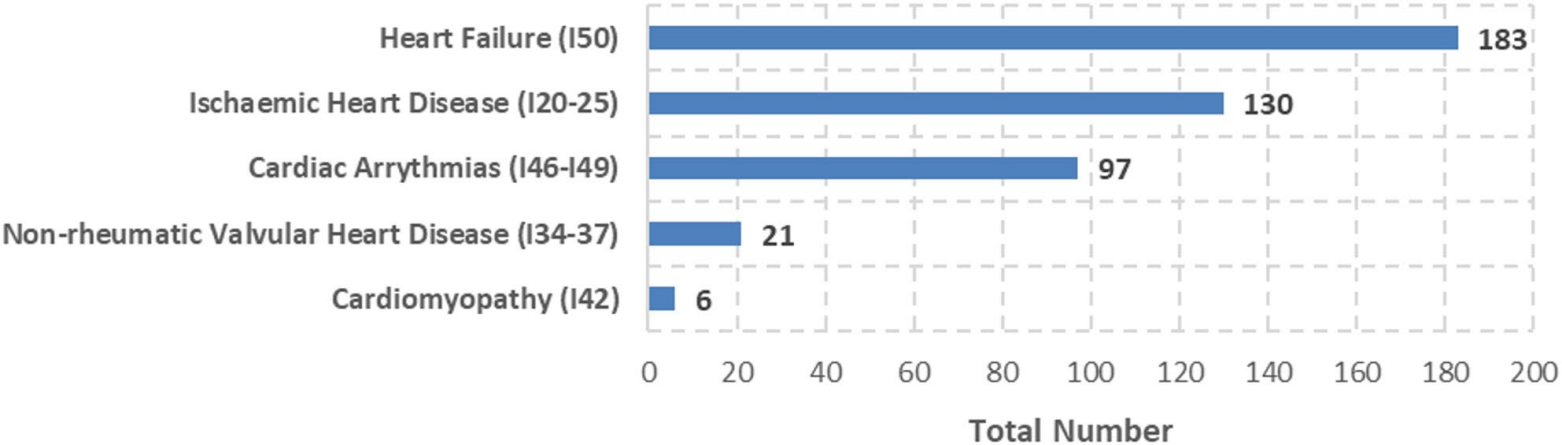
Number of studies reporting patient-reported outcomes and experiences by CVD according to ICD-10 diagnoses (multiple entries per study possible)

Most studies focused on HF-patients (*n* = 183; 47%). In the majority of studies, the HF-subtype was not specified (*n* = 137; 75%). Of the 46 studies that differentiated between subtypes, the most frequently analysed was HF with reduced ejection fraction (HFrEF; *n* = 37; 80%), due to their prevalence in the general population, availability of treatment options and ongoing research on its management and outcomes. Non-rheumatic valvular heart diseases (*n* = 21; 5%) and cardiomyopathies (*n* = 6; 2%) were only rarely assessed with regard to PROs and PREs (Fig. 2).

PROs and PREs were particularly frequently reported in patients with HF (n_PRO_=153, 44%; n_PRE_=72, 39%), ischemic heart disease (n_PRO_=95, 27%; n_PRE_=63, 34%), and cardiac arrythmias (n_PRO_=76, 22%; n_PRE_=42, 23%). In patients with non-rheumatic valvular heart disease, the proportion of PROs measured was substantially higher compared to PREs (n_PRE_=5; n_PRO_=20).

#### Comorbidities

To further describe the setting of the included studies (objective A), CVD-relevant comorbidities among study populations were identified in addition to study type, purpose and population. More than two-thirds of the studies assessed comorbidities relevant to CVD in addition to the underlying disease (*n* = 276; 71%). The most common comorbidities examined were diabetes (*n* = 222; 57%), additional CVDs (*n* = 195; 50%) and hypertension (*n* = 187; 48%). However, only a few studies assessing diabetes differentiated between types of diabetes (*n* = 15; 7%). Comorbidities such as anxiety disorders (*n* = 12; 3%), obesity (*n* = 46; 12%), depression (*n* = 53; 14%), and dyslipidaemia (*n* = 59; 15%) were rarely assessed, despite their relevance.

### Patient-reported outcome measures

#### Measurement instruments

**Fig. 3:**
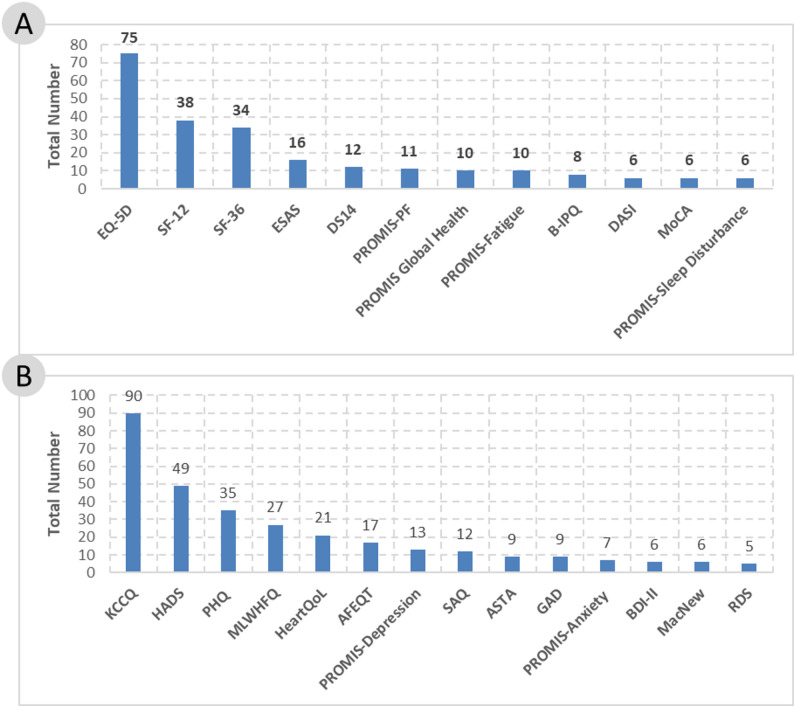
(**A**) Most frequently used generic patient-reported outcome measures; (**B**) most frequently used disease-specific patient-reported outcome measures

A total of 140 unique, validated PROMs were identified. Of these, 127 were retrieved through the PubMed search, while an additional 13 were added through the ClinicalTrials.gov analysis (see Appendix 3). Most PROMs were generic PROMs (*n* = 87; 62%), which are not focused on a specific disease and can be used for a wide variety of interventions [24]. Despite the large number of generic PROMs, disease-specific PROMs were mostly used in the identified studies. These PROMs focus on specific diseases and capturing more specific aspects related to them [24]. Of the 309 studies measuring PROs, 248 (80%) used disease-specific questionnaires and 195 (63%) used generic ones. However, only 26 of the 140 identified PROMs (19%) were used in more than five studies (Fig. 3). Thereby, the most used generic PROMs were the EQ-5D of EuroQol (incl. EQ-VAS separately used), the Short Form-12 Health Survey (SF-12) and the SF-36, which measure QoL, health status and symptom burden. Commonly used disease-specific PROMs include the Kansas City Cardiomyopathy Questionnaire (KCCQ), Hospital Anxiety and Depression Scale (HADS), Patient Health Questionnaire (PHQ) and Minnesota Living with Heart Failure Questionnaire (MLWHFQ), which measure QoL, symptoms of anxiety and depressive symptoms.

In the 309 studies measuring PROs, 362 measurement instruments were used (Table 4). As only standardised and validated measurement instruments were assessed for measuring PROs, questionnaires (*n* = 301; 83%) were used much more frequently than interviews (*n* = 53; 15%). Most of these were paper-based questionnaires (*n* = 117; 39%). However, electronic questionnaires accessible online via a web browser, app or tablet were also in use (*n* = 42; 14%). It can also be seen that, from 2017 onwards, PROMs were recorded electronically more frequently, particularly in studies aiming to assess adherence and test the feasibility of PRO/PRE assessments, as well as in studies seeking to develop and validate questionnaires. For eight PROMs, the type of measuring was unclear.

**Table 4:** Format for measuring pros

	Format of measurement	Count	Proportion
**Questionnaire**	Not mentioned / unclear	135	45%
	App-based questionnaire	6	2%
	Electronic questionnaire (no further information)	10	3%
	Electronic questionnaire via a tablet	9	3%
	E-mailed questionnaire	7	2%
	Paper-based questionnaire	117	39%
	Web-based / online questionnaire	17	6%
**Interview**	Not mentioned / unclear	6	11%
	Face-to-face interview	13	25%
	Telephone interview	34	64%

#### Patient-reported outcomes

**Fig. 4:**
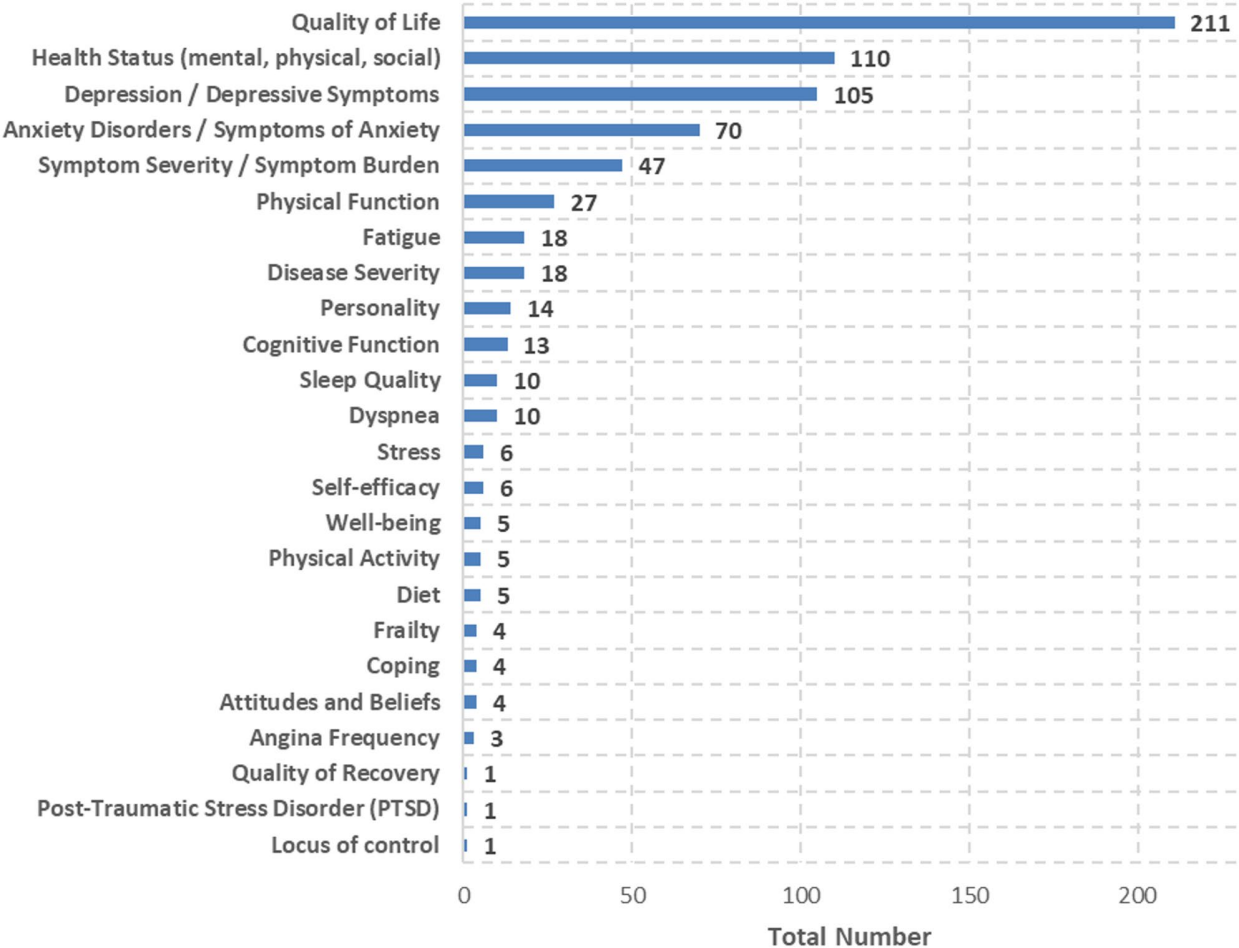
Total number of PROs measured grouped by PRO category

A total of 24 groups of PROs were identified (Fig. 4). QoL was the most frequently assessed PRO, included in 211 out of 309 studies (68%). Nevertheless, health status (*n* = 110; 36%) and depression or depressive symptoms (*n* = 105; 34%) were also assessed in approximately one third of the studies.

**Fig. 5:**
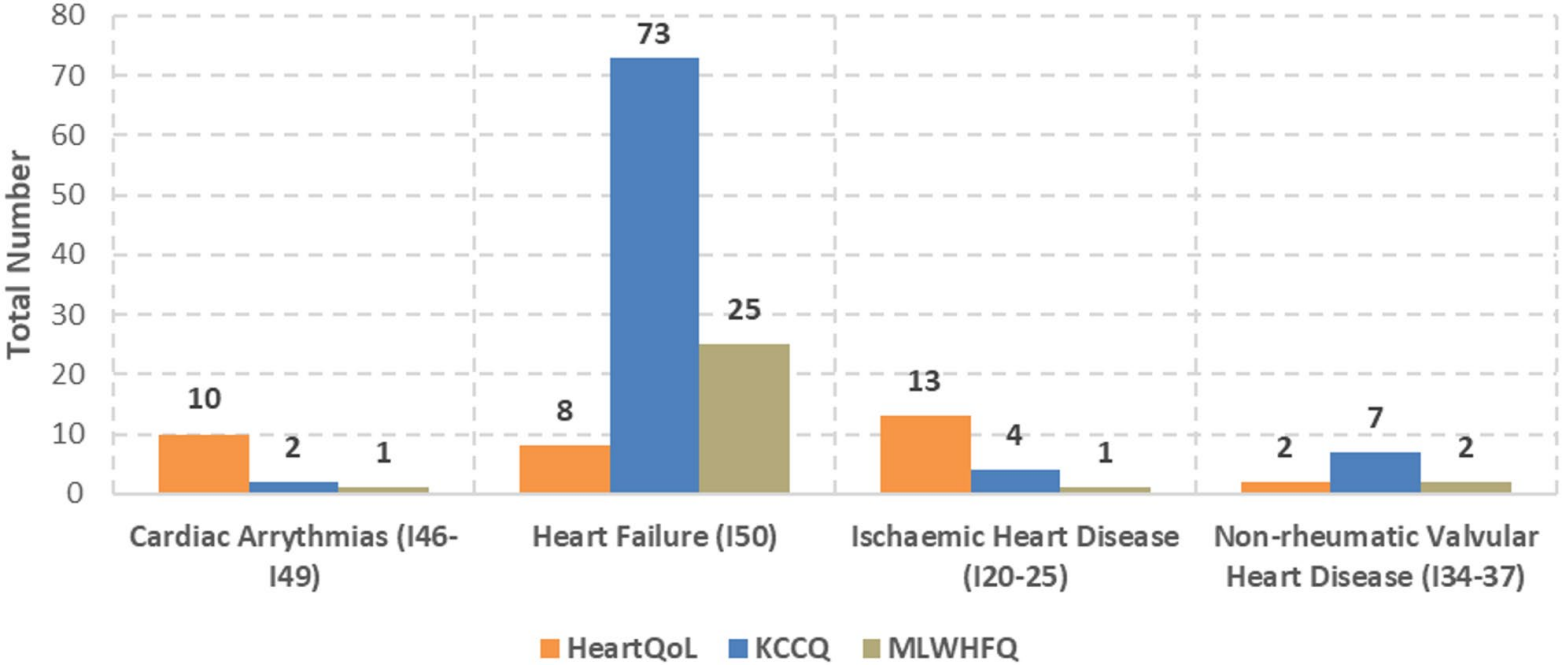
Number of disease-specific questionnaires used to measure QoL grouped by CVDs

QoL was assessed 325 times across the 309 included studies. Of these assessments, 203 (62%) used disease-specific PROMs. Depression/depressive symptoms were measured 67 times, with disease-specific PROMs applied in 62 of these assessments (93%). Health status, on the other hand, was mostly measured using generic PROMs (*n* = 124; 82%). For measuring QoL, 17 different disease-specific questionnaires were identified. Thereby, KCCQ was used most frequently (*n* = 76; 46%), followed by MLWHFQ (*n* = 27; 16%) and HeartQoL (*n* = 20; 12%). KCCQ was used in different versions, mostly the KCCQ-23 (*n* = 53, 70%) and the short version KCCQ-12 (*n* = 22; 29%). Although the KCCQ was developed specifically to measure QoL in chronic HF and HeartQoL for ischaemic heart disease (IHD), both have been widely used in other CVDs (Fig. 5). In contrast, MLWHFQ, being developed to measure QoL in chronic HF-patients, is rarely used for other CVDs. Of the generic PROMs used, EQ-5D (excl. EQ-VAS) was by far the most frequently used questionnaire (*n* = 67; 74%), followed by the Patient-Reported Outcomes Measurement Information System (PROMIS) Global Health (*n* = 10; 11%). Regarding the EQ-5D version, the long version was slightly preferred (EQ-5D-5 L; *n* = 30; 45%) to the short version (EQ-5D-3 L; *n* = 22; 33%). Looking more closely at the CVDs in which QoL was recorded, it is noticeable that disease-specific PROMs (*n* = 117; 71%) are used more frequently than generic questionnaires for HF. For IHDs, on the other hand, the data is mixed (n_disease−specific_=36; 45%).

Five different disease-specific PROMs were identified for measuring depression and depressive symptoms. PHQ-Depression (*n* = 35; 56) and PROMIS-Depression (*n* = 13; 21%) were the most widely used. In most cases, the long version of the PHQ (PHQ-9) was used (*n* = 22; 63%), followed by the shortest version PHQ-2 (*n* = 8; 23%). Depression was only assessed in patients with HF, IHDs, and cardiac arrythmias.

Health status was analysed in four categories: general health status (*n* = 100; 91%), mental health (*n* = 5; 4%), physical health (*n* = 2; 2%), and social health (*n* = 3; 3%). Ten different generic PROMs were identified for measuring health status. The SF in its various versions was by far the most frequently used (*n* = 71; 57%). It was used for all CVDs, but most frequently for IHD (*n* = 28; 31%) and cardiac arrhythmias (*n* = 26; 29%). No clear trend emerged in the use of either the SF-12 (*n* = 37; 52%) or the SF-36 (*n* = 32; 45%). Regarding disease-specific PROMs, it becomes clear that there are only a small number of validated, disease-specific PROMs that measure health status. This is particularly evident given that no disease-specific questionnaires could be identified for cardiac arrhythmias, cardiomyopathy, or non-rheumatic valvular heart disease.

### Patient-reported experience measures

#### Measurement instruments

**Fig. 6:**
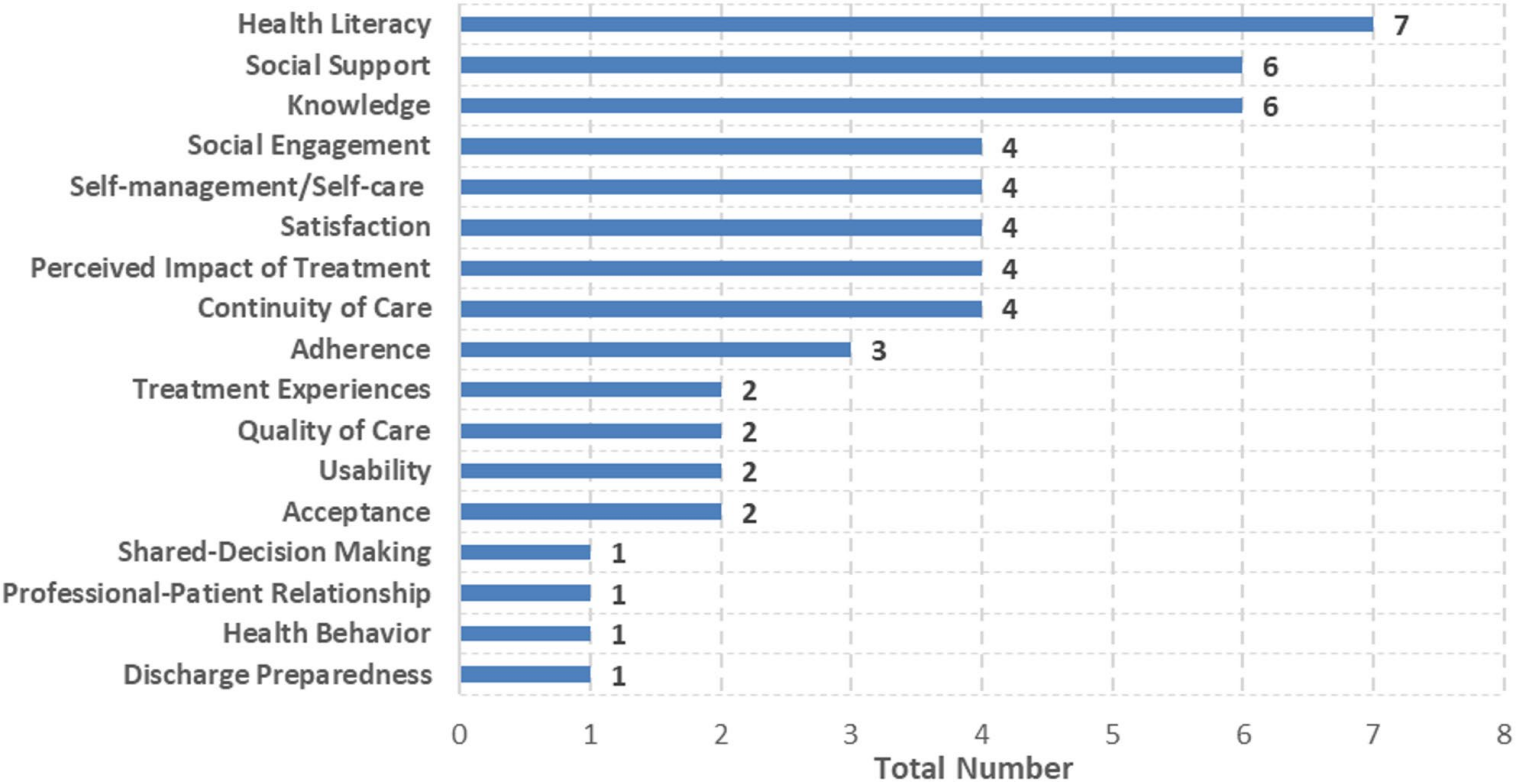
Number of validated PREMs for measuring PREs

A total of 166 different PREMs were identified by searching PubMed and ClinicalTrials.gov (see Appendix 3 for standardised PREMs). Of these, more than half were self-developed questionnaires or interview guides (*n* = 94; 57%). Only a third were validated PREMs (*n* = 56; 34%). Most of these exist to evaluate health literacy, social support or knowledge (Fig. 6). Among other things, there were no validated questionnaires to evaluate end-of-life preferences, expectations, patient empowerment, or patient involvement in patients with CVDs. Furthermore, validated PREMs often do not focus on a specific disease (*n* = 40; 71%). Of those that do, most addressed CVDs in general (*n* = 7) or HF (*n* = 5).

Of the 159 studies measuring PREs, at least 65 (41%) used validated questionnaires, while the majority used self-developed PREMs (*n* = 94; 59%) or standardized, non-validated ones (*n* = 19; 12%). A combination of self-developed and validated PREMs was rare (*n* = 9; 6%). Most studies used either self-developed (*n* = 49; 75%) or validated (*n* = 83; 88%) PREMs. Most of validated questionnaires were used in patients with HF (*n* = 37; 47%), followed by IHD (*n* = 20; 25%) and cardiac arrythmias (*n* = 19; 24%).

Despite the frequent inclusion of PREMs in qualitative studies, questionnaires were used in 127 out of 194 cases (66%) to measure PRE (Table 5). As with PROMs, most of these were paper-based (*n* = 56; 44%). However, a higher proportion of electronic questionnaires were accessible online via a web browser, an app or a tablet than for PROMs (*n* = 27; 22%). PRE were collected electronically, especially when the study aimed to assess feasibility of new PRO/PRE assessments and develop new questionnaires/assessment tools. From 2022 onwards, electronic measurement of PREs took place just as frequently, if not more frequently, than paper-based measurement.

**Table 5:** Format for measuring pres

	Format of measurement	Count	Proportion
Questionnaire	Not mentioned / unclear	43	34%
	App-based questionnaire	3	2%
	Electronic questionnaire (no further information)	6	5%
	Electronic questionnaire via a tablet	2	2%
	E-mailed questionnaire	1	1%
	Paper-based questionnaire	56	44%
	Web-based / online questionnaire	16	13%
Interview	Not mentioned / unclear	9	16%
	Face-to-face interview	26	46%
	Telephone interview	22	39%
Workshop	Face-to-face workshop	4	67%
	Online workshop	2	33%

Bearing in mind that non-standardised PROMs were excluded, the proportion of interviews used was significantly higher than that for PROMs (29% for PREMs vs. 15% for PROMs). Furthermore, workshops were also implemented to measure PRE (*n* = 6; 3%). In most cases, these were face-to-face workshops (*n* = 4) designed to gather information on treatment experiences, patient empowerment, adherence, and facilitators and barriers to participate in treatment.

#### Patient experience data

**Fig. 7:**
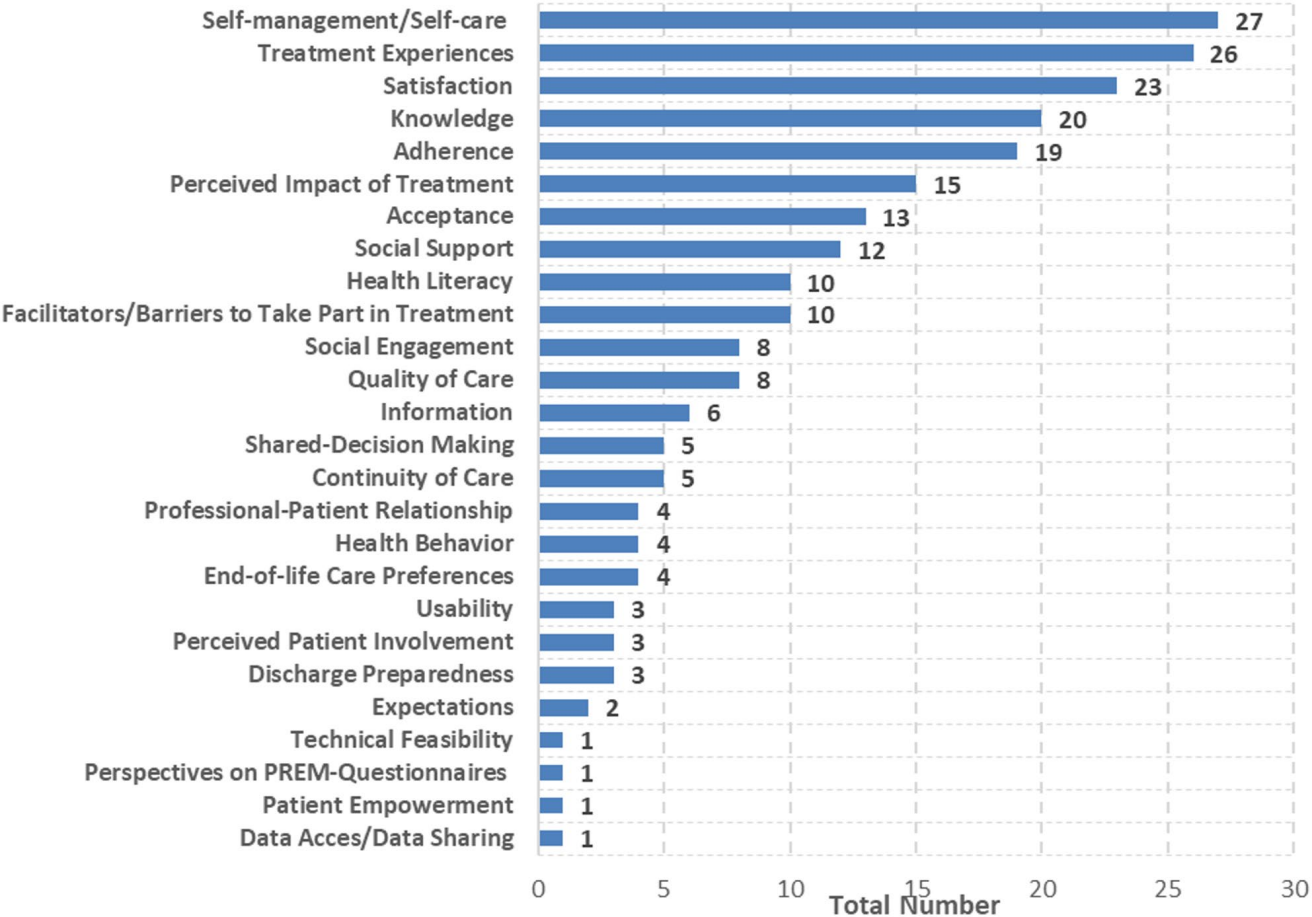
Total number of patient-reported experiences measured

A total of 26 groups of PREs were identified (Fig. 7). In most cases, self-care (*n* = 27; 17%), treatment experiences (*n* = 26; 16%), overall satisfaction (*n* = 23; 14%), knowledge (*n* = 20; 13%), and adherence (*n* = 19; 12%) were assessed. In contrast to PROs, no particular PRE was particularly common.

In almost two thirds of cases, self-care was measured using one of the four identified validated questionnaires (*n* = 17; 63%). Three of these questionnaires were disease-specific (HF = 2; diabetes = 1). Most commonly, the Self-Care of Heart Failure Index (SCHFI) (*n* = 8) and the European Heart Failure Self-Care Behavior Scale (EHFScB) (*n* = 6) were used. In addition, the non-validated but standardized Response to Symptoms Questionnaire (RSQ) was used in 5 studies (*n* = 5; 19%). In self-developed questionnaires and interview guides used to measure self-care, patients were asked about their self-care behaviour (*n* = 6), for example diet and medication intake, health education and knowledge of their disease (*n* = 3), and communication with their healthcare professionals (*n* = 3).

Treatment experiences were almost always measured using self-developed questionnaires (*n* = 23; 88%). Only two validated PREMs were identified for measuring treatment experience: the non-disease-specific Canadian Patient Experiences Survey-Inpatient Care (CPES-IC) and the Sense of Security in Care-Patients’ Evaluation (SEC-P). Both were used in only one study. Similarly, the non-validated but standardized Generic Short Patient Experiences Questionnaire (GS-PEQ) was only used in one study. Various aspects were covered in self-developed PREMs. Patients were mostly asked about their experiences of care coordination (*n* = 9), communication (*n* = 9) and the professional-patient relationship (*n* = 7), their engagement in the care process, including shared decision-making (*n* = 6), their perception of the quantity, quality and provision of information (*n* = 9), and the quality of care in general (*n* = 6).

In more than a third of the studies, satisfaction was measured using validated PREMs. (*n* = 9; 38%). Thereby, four validated questionnaires were identified. Half of these were disease-specific for different CVDs. The most used questionnaires were the Anti-Clot Treatment Scale (ACTS; *n* = 4) and the Perception of Anticoagulant Treatment Questionnaire (PACT-Q; *n* = 3). Just as satisfaction with a treatment is a rather unspecific PRE, the aspects used to measure satisfaction are also rather unspecific. For example, simple questions were often asked about general satisfaction with the treatment or quality of care in general (*n* = 8). Furthermore, satisfaction was often assessed using self-developed PREMs in the context of hospital care, evaluating the experiences of patients in general (*n* = 7). However, specific aspects were also assessed albeit less frequently. These included the coordination of care (*n* = 4) and communication with healthcare professionals (*n* = 4).

Only half of the studies measuring knowledge used standardised questionnaires, either validated or non-validated (*n* = 11; 55%). Almost all of these were disease-specific for patients with HF or AF (6 out of 7). The Mishel Uncertainty Illness Scale (MUIS) is the only generic, validated questionnaire designed to measure knowledge. The Dutch Heart Failure Knowledge Scale (DHFKS) and the Jessa Atrial Fibrillation Knowledge Questionnaire (JAKQ) were the only PREMs used in more than one study. Self-developed questionnaires and interview guides were mostly used to measure knowledge in patients with cardiac arrhythmias (7 out of 9). Overall, knowledge of the underlying disease (*n* = 5) and treatment (*n* = 4) was assessed.

Despite validated PREMs being available for measuring adherence, only three of the 19 studies reporting on adherence used a validated questionnaire. Each of these studies used a different questionnaire: (1) Medical Outcome Study Specific Adherence Scale (MOSSAS), (2) Revised Heart Failure Compliance Questionnaire (RHFCQ), and (3) Adherence to Refills and Medications Scale (ARMS). The most commonly used was the Morisky Medication Adherence Scale (MMAS; *n* = 5), which is a standardised but not validated instrument for evaluating medication compliance in patients with chronic diseases. In 58% of studies (*n* = 11), self-developed questionnaires or interview guides were employed. These were mainly designed to evaluate medication adherence (*n* = 7), but also to evaluate adherence to lifestyle changes (*n* = 2), general adherence (*n* = 1) and adherence to self-monitoring activities (*n* = 1).

## Discussion

The findings show that PROMs are used far more often than PREMs when evaluating the quality of care for CVDs. Most studies used disease-specific, validated PROMs. Self-developed and non-validated instruments were commonly used to measure PREs. Thereby, it should be noted that, by definition, only validated PROMs were included in this review. This methodological decision likely explains the discrepancy observed between the number of studies assessing PROs and those assessing PREs, particularly regarding qualitative study designs. Moreover, standardised quantitative PROMs are primarily used to enable large-scale comparison and aggregation, for example in the evaluation of therapies and healthcare services, and in the assessment of regular care. Thereby, the reliability of validated multi-item PROMs is similar to that of clinical measures [25]. So-called ‘single-transitional items’ were used in only a few cases. This means that individual questions were incorporated into interview guides or self-developed questionnaires to gather information about patients’ health from their perspective.

The much wider use of PROMs compared to PREMs can be explained by an early consensus about PROMs as valuable additional indicators of the quality of healthcare services and treatments, alongside traditional indicators. The PROMIS project was launched in 2004 with the support of the National Institutes of Health (NIH) in order to improve communication between physicians and patients and thus achieve a considerable benefit for patients [26]. Today, a large number of generic PROMIS measures exist for the assessment of various PROs, such as depression, fatigue, cognitive function, and pain quality [27]. In addition, PROMs were already included in national standards in 2008. For example, the NHS Contract for Acute Services standard includes requirements that healthcare providers must also report on PROMs [28]. Thereby, the collection of PROMs data has been made mandatory for non-Foundation Trust NHS Acute Trusts. Overall, the number of trials that include at least one PRO as an additional endpoint has been increasing since 2017.

Whereas the standardized measurement of PREs also began back in 2002 with the HCAHPS Survey, a national, standardized survey of patients perspective in hospitals [29], only in 2011 the NHS National Quality Board became one of the first to lay a foundation for the definition and measurement of PRE with the NHS Patient Experience Framework, expressing how critical the collection of PREs is for healthcare services. Also, between 2006 and 2016, OECD countries focused their work more intensively on measuring PREs, achieving great success in 2017, particularly with regard to collection and international comparisons of PRE on a national level [30].

The fact that many self-developed PREMs are still in use, often as the only instrument in a study, highlights an important shortcoming: Whereas PROMs benefit from a wide range of validated, often disease-specific measures, the development and validation of PREMs is lagging significantly behind [31]. The result is a considerable heterogeneity with respects of patient experience measured, and in the methods used to measure them. This lack of standardisation complicates comparisons across studies and settings. In the specific context of CVD, this is particularly problematic because constructs such as shared decision-making, end-of-life preferences, or continuity of care are highly relevant for patients with chronic and progressive diseases, yet no validated CVD-specific PREMs exist to capture them. As with PROMs, contemporary literature is increasingly calling for the rigorous development and standardisation of PREMs [32]. In addition, the combination of qualitative and quantitative materials, such as surveys, online assessments, in-depth interviews, focus groups and patient panels, as recommended in the literature, was rarely employed to gain a better understanding of the depth and complexity of patient experiences [31].

A further implication of our findings relates to the choice between generic and disease-specific measures. Generic PROMs and PREMs facilitate comparisons across diseases and healthcare systems; however, they may fail to capture clinically significant nuances specific to CVD populations (e.g. the disease burden associated with arrhythmias or the psychosocial impact of heart failure). Conversely, disease-specific instruments offer a more comprehensive range of data but compromise the ability to make meaningful comparisons. Consequently, clinicians and researchers in cardiology are required to judiciously balance the trade-off between comparability and disease-specific sensitivity when selecting measures.

The type of administration was not reported for almost half of the questionnaires. Nevertheless, available data and trends from the literature suggest that paper-based assessment continues to predominate, despite the increasingly recognised advantages of electronic data capture, including improved data quality, patient convenience and real-time analysis [4, 33]. It is thus probable that this review underestimates the true proportion of paper-based assessments. Although the use of electronic PROM and PREM collection is growing, it is still far from universal and is often poorly documented in the literature. For CVD populations, where frequent follow-up and monitoring are required, the lack of widespread digital implementation may hinder integration into routine care and reduce accessibility for certain patient groups. Given the opportunities for streamlining data collection and integrating it into electronic health records, there is clearly a need for more structured reporting and for electronic instruments to be adopted [34].

### Limitations

A key limitation of this work is that the quality of the included studies was not systematically assessed. Important aspects such as study design (e.g., randomization, blinding), sample size, and quality of reporting were not evaluated, which means that both high- and low-quality studies may have contributed equally to the findings [35]. With this the ability to draw strong conclusions about the validity and generalizability of the results is restricted.

Due to the high number of publications retrieved, reviews, meta-analyses and systematic syntheses were excluded at the full-text screening stage. While this may have resulted in valuable information being lost, such as insights into the comparative utility or long-term implementation of PROMs and PREMs, it did allow for a more focused analysis of primary studies and mitigated the risk of double-counting data.

Another limitation concerns the response rate of patients included in the studies, which limits the validity, reliability and real-world applicability of certain measures, particularly with regard to their acceptability and feasibility in clinical practice [36]. As the response rate was extracted where possible but could not be analysed due to a lack of information within the examined studies, it was not possible to evaluate the potential impact of non-response bias, or to obtain an indication of the quality of specific PROMs and PREMs. There was also no direct assessment of the quality or suitability of questionnaires and interview guides for specific diseases or study purposes. Analysis of individual PROMs and PREMs was limited to frequency of use in different studies. Consequently, the results reflect utilisation patterns rather than critical evaluations or recommendations regarding the appropriateness of specific measures.

Only validated measures were included for assessing PROs. However, for measuring PREs, both validated and non-validated measures were considered, as it was expected that the number of validated PREMs for CVD is still relatively small. This resulted in an asymmetry in the methodological approach to identifying PROMs and PREMs. While this ensured comprehensive coverage of existing PREMs, it may also have resulted in the inclusion of more non-validated and potentially less reliable tools. Furthermore, standardised but not yet validated PROMs with high future usage potential may not have been considered. However, it seems that the number is low, as only 47 of the 879 full texts screened were removed due to the absence of a validated PROM.

A further limitation relates to the distribution of CVD types represented in the included studies. HF was by far the most frequently investigated condition (47% of all included studies). This aligns with the findings of other reviews, reflecting its epidemiological relevance and the focus of instrument development [37, 38]. Many widely used PROMs are disease-specific for HF, such as the KCCQ. As a result, the high prevalence of HF in the study population is likely to influence the instruments and outcomes that are most frequently measured. This concentration may introduce selection bias. While the results are generalisable to HF populations, their applicability to other cardiovascular conditions, such as arrhythmias, valvular disease or ischaemic heart disease, is considerably more limited. Therefore, the evidence base for PROMs and PREMs in non-HF cardiovascular diseases remains limited. This should be considered when interpreting the findings of this scoping review.

Another limitation is the exclusion of certain CVDs, such as those secondary to birth defects, neurological or rheumatic causes, as well as patients at risk without manifest disease. Consequently, the generalisability of the findings is limited to the more prevalent adult CVDs and does not apply to the full range of cardiovascular conditions.

Finally, this review is limited by the exclusion of studies addressing individuals at an early risk of developing CVD (stage 1). Although this approach ensured a consistent focus on patients with manifest disease, it limits the applicability of the findings to preventive contexts. Future research could therefore expand the scope by mapping PROMs and PREMs used in risk prevention and early intervention settings.

## Conclusions

Motivated by the growing awareness that capturing patients’ perspectives is crucial to improving healthcare quality, this review provides a valuable, comprehensive overview of the current state of PROMs and PREMs in CVD research and care. The findings reveal that PROMs are utilised more frequently than PREMs, with QoL, health status, and depressive symptoms being the most frequently assessed PROs. Despite numerous existing measures, there is significant variation in the selection and use of PROMs and PREMs. Those reflecting PREs are particularly underrepresented, often being measured with non-validated or even non-standardised instruments. This highlights the need for further standardisation and longitudinal assessment of measurement approaches. Standardising measurement instruments and their application would enhance comparability between studies, support patient-centred care and ultimately improve outcomes for individuals living with CVD.

It also emphasises the importance of developing validated PREMs that are tailored to cardiovascular populations across the care continuum. To achieve this, future research should be based on well-established methodological frameworks, such as the COSMIN guidelines, to ensure the rigorous development and validation of CVD-specific PREMs. Particular attention should be given to domains for which no validated instruments currently exist, such as end-of-life preferences and continuity of care.

## Supplementary Information

Below is the link to the electronic supplementary material.

Supplementary Material 1

Supplementary Material 2

Supplementary Material 3

Supplementary Material 4

## Data Availability

The datasets used and/or analysed during the current study are available from the corresponding author on reasonable request.

## Abbreviations

ACTS: Anti-Clot Treatment Scale
ARMS: Adherence to Refills and Medications Scale
CAD: Coronary artery disease
CPES-IC: Canadian Patient Experiences Survey - Inpatient Care
CVD: Cardiovascular disease
DHFKS: Dutch Heart Failure Knowledge Scale
EHFScB: European Heart Failure Self-Care Behavior Scale
GS-PEQ: Generic Short Patient Experiences Questionnaire
HADS: Hospital Anxiety and Depression Scale
HF: Heart failure
iCARE4CVD: Individualised care from early risk of cardiovascular disease to established heart failure
IHD: Ischaemic heart disease
JAKQ: Jessa Atrial Fibrillation Knowledge Questionnaire
KCCQ: Kansas City Cardiomyopathy Questionnaire
MLWHFQ: Minnesota Living with Heart Failure Questionnaire
MMAS: Morisky Medication Adherence Scale
MOSSAS: Medical Outcome Study Specific Adherence Scale
MUIS: Mishel Uncertainty Illness Scale
PACT-Q: Perception of Anticoagulant Treatment Questionnaire
PHQ: Patient Health Questionnaire
PRE: Patient-reported experiences
PREM: Patient-reported experience measure
PRO: Patient-reported outcome
PROM: Patient-reported outcome measure
PROMIS: Patient-Reported Outcomes Measurement Information System
RCT: Randomized controlled trial
RHFCQ: Revised Heart Failure Compliance Questionnaire
RSQ: Response to Symptoms Questionnaire
SCHFI: Self-Care of Heart Failure Index
SEC-P: Sense of Security in Care - Patients’ Evaluation
