# Supplementary material for: Patient-reported outcome and experience measures in cardiovascular disease: a scoping review as part of iCARE4CVD
Source: J Patient Rep Outcomes. 2025 Dec 9;9:141. doi: 10.1186/s41687-025-00980-4 (PMC12696235; doi:10.1186/s41687-025-00980-4)

**Appendix 1: Search strings**

**PubMed**

(“Cardiovascular Disease*”[tiab] OR Cardiovascular Diseases[MeSH Terms] OR “Cardiac Disease*”[tiab] OR “Cardiac Disorder*”[tiab] OR “Heart Disease*”[tiab] OR “Heart Disorder*”[tiab] OR “Cardiac Event*”[tiab] OR “Vascular Disease*”[tiab]) **AND**

(“Health Care Survey*”[tiab] OR “Healthcare Survey*”[tiab] OR Health Care Surveys[MeSH Terms] OR “Patient Reported Outcome*”[tiab] OR “Patient-Reported Outcome*”[tiab] OR Patient Reported Outcome Measures[MeSH Terms] OR PROM[tiab] OR “Patient Outcome Assessment”[tiab] OR “Patient Reported Experience*”[tiab] OR “Patient-Reported Experience*”[tiab] OR PREM[tiab] OR Patient Perspective[tiab] OR “Patient Journey*”[tiab])

**ClinicalTrials.gov**


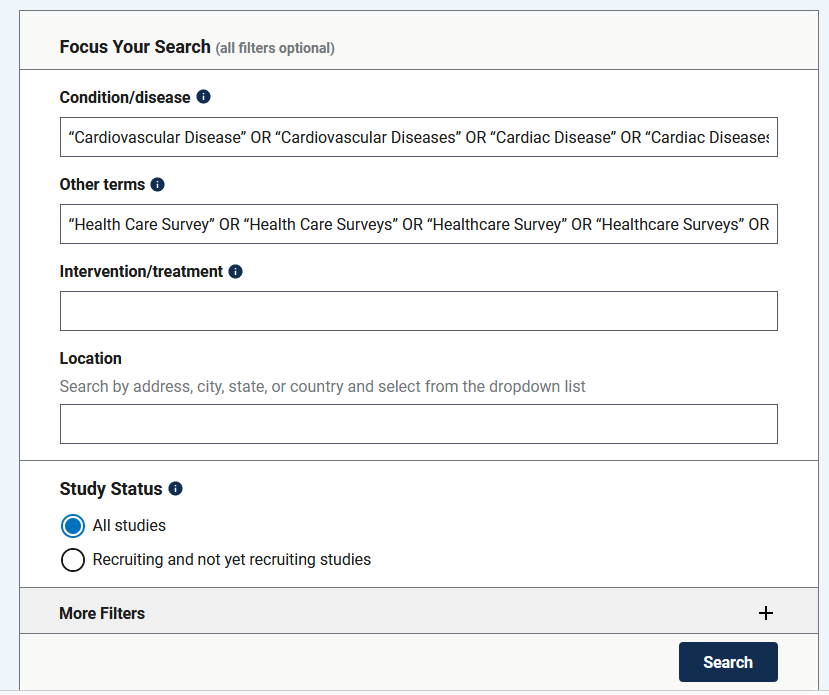

Supplement: Supplementary file 1 — Supplementary Material 1 [file 41687_2025_980_MOESM1_ESM.docx]
