# Supplementary material for: Patient-reported outcome and experience measures in cardiovascular disease: a scoping review as part of iCARE4CVD
Source: J Patient Rep Outcomes. 2025 Dec 9;9:141. doi: 10.1186/s41687-025-00980-4 (PMC12696235; doi:10.1186/s41687-025-00980-4)
Supplement: Supplementary file 2 — Supplementary Material 2 [file 41687_2025_980_MOESM2_ESM.docx]

**Appendix 2: List of analysed publications**

| Nr. | Author | Title | Year | Journal | Volume | Pages/ Article Number |
| --- | --- | --- | --- | --- | --- | --- |
| 1 | Abdallah, M. S. et al. | Quality of life after PCI vs CABG among Patients with Diabetes and Multivessel CAD: Results from the FREEDOM Trial | 2013 | JAMA | 310(15) | 1581-1590 |
| 2 | Abdelaziz, H. K. et al. | Quality of Life Assessment in Patients Undergoing Trans-Catheter Aortic Valve Implantation Using MacNew Questionnaire | 2022 | The American Journal of Cardiology. | 164 | 103-110 |
| 3 | Abraham, W. T. et al. | Effect of empagliflozin on exercise ability and symptoms in heart failure patients with reduced and preserved ejection fraction, with and without type 2 diabetes | 2021 | European Heart Journal | 42 | 700-710 |
| 4 | Ahmad, F. S. et al. | Development and Initial Validation of the PROMIS®-Plus-HF Profile Measure | 2019 | Circulation. Heart Failure | 12(6) | e005751 |
| 5 | Ahmad, F. S. et al. | The development and initial validation of the PROMIS®+HF-27 and PROMIS+HF-10 profiles | 2022 | ESC Heart Failure | 9 | 3380-3392 |
| 6 | Alarouri, H. S. et al. | Prognostic Value of Patient-Reported Outcomes in Predicting Long-Term Mortality After Transcatheter Aortic Valve Replacement | 2023 | Journal of the American Heart Association | 12 | e030383 |
| 7 | Ali-Ahmed, F. et al. | Shared decision-making in atrial fibrillation: patient-reported involvement in treatment decisions | 2020 | European Heart Journal - Quality of Care and Clinical Outcomes | 6 | 263-272 |
| 8 | Alonso, W. W. et al. | The HEART Camp Exercise Intervention Improves Exercise Adherence, Physical Function, and Patient-Reported Outcomes in Adults with Preserved Ejection Fraction Heart Failure | 2022 | Journal of cardiac failure | 28(3) | 431-442 |
| 9 | Amara, W. et al. | Patients' attitude and knowledge about oral anticoagulation therapy: results of a self-assessment survey in patients with atrial fibrillation conducted by the European Heart Rhythm Association | 2016 | Europace | 18 | 151-155 |
| 10 | Ammenwerth, E. et al. | Evaluation of an Integrated Telemonitoring Surveillance System in Patients with Coronary Heart Disease | 2015 | Methods of information in medicine | 54(5) | 388-397 |
| 11 | Andersen, K. S. et al. | Correlation Between Exercise Capacity and Quality of Life in Patients With Cardiac Disease | 2018 | Journal of Cardiopulmonary Rehabilitation and Prevention | 38 | 297-303 |
| 12 | Anderson, D. R. et al. | Population-based evaluation of the management of antithrombotic therapy for atrial fibrillation | 2005 | The Canadian journal of cardiology | 21(3) | 257-266 |
| 13 | Anderson, H. et al. | The concerns of patients under palliative care and a heart failure clinic are not being met | 2001 | Palliative Medicine | 15 | 279-286 |
| 14 | Arnold, S. V. et al. | Patient-reported vs. physician-estimated symptoms before and after transcatheter aortic valve replacement | 2022 | European Heart Journal - Quality of Care and Clinical Outcomes | 8 | 161-168 |
| 15 | Auensen, A. et al. | Patient-reported outcomes after referral for possible valve replacement in patients with severe aortic stenosis | 2018 | European Journal of Cardio-Thoracic Surgery | 53 | 129-135 |
| 16 | Auld, J. P. et al. | Device-detected congestion is associated with worse patient-reported Outcomes in Heart Failure | 2019 | Heart Lung | 48(3) | 208-214 |
| 17 | Bachmann, J. M. et al. | Perceived Health Competence Predicts Health Behavior and Health-Related Quality of Life in Patients with Cardiovascular Disease | 2016 | Patient education and counseling | 99(12) | 2071-2079 |
| 18 | Badin, A. et al. | Patients' and Physicians' Perceptions Regarding the Benefits of Atrial Fibrillation Ablation | 2017 | Pacing and clinical electrophysiology: PACE | 40(4) | 362–371 |
| 19 | Baik, D. et al. | Measuring health status and symptom burden using a web-based mHealth application in patients with heart failure | 2019 | European Journal of Cardiovascular Nursing | 18(4) | 325-331 |
| 20 | Baker, D. W. et al. | Differences in Education, Knowledge, Self-Management Activities, and Health Outcomes for Patients With Heart Failure Cared for Under the Chronic Disease Model: The Improving Chronic Illness Care Evaluation | 2005 | Journal of Cardiac Failure | 11(6) | 405-413 |
| 21 | Baker, D. W. et al. | A Telephone Survey to Measure Communication, Education, Self-Management, and Health Status for Patients With Heart Failure: The Improving Chronic Illness Care Evaluation (ICICE) | 2005 | Journal of Cardiac Failure | 11(1) | 36-42 |
| 22 | Bakhai, A. et al. | Patient perspective on the management of atrial fibrillation in five European countries | 2013 | BMC Cardiovascular Disorders | 13 | 108 |
| 23 | Bakitas, M. A. et al. | Effect of an Early Palliative Care Telehealth Intervention vs Usual care on Patients With Heart Failure | 2020 | JAMA Internal Medicine | 180(9) | 1203-1213 |
| 24 | Bamgbade, B. A. et al. | Differences in Perceived and Predicted Bleeding Risk in Older Adults With Atrial Fibrillation: The SAGE-AF Study | 2021 | Journal of the American Heart Association | 10 | e019979 |
| 25 | Barmano, N. et al. | Predictors of improvement in arrhythmia-specific symptoms and health-related quality of life after catheter ablation of atrial fibrillation | 2019 | Clinical Cardiology | 42(2) | 247-255 |
| 26 | Barsky, L. et al. | Sex-based differences in remote monitoring of biometric, psychometric and biomarker indices in stable ischemic heart disease | 2022 | Biology of sex differences | 13(1) | 15 |
| 27 | Beckie, T. M. et al. | A Mobile Health Behavior Change Intervention for Women With Coronary Heart Disease | 2024 | Journal of Cardiopulmonary Rehabilitation and Prevention | 44 | 40-48 |
| 28 | Bekelman, D. B. et al. | Effect of a Collaborative Care Intervention vs Usual Care on Health Status of Patients With Chronic Heart Failure | 2018 | JAMA Internal Medicine | 178(4) | 511-519 |
| 29 | Benzer, W. et al. | Short-term patient-reported outcomes after different exercise-based cardiac rehabilitation programmes | 2007 | Eur J Cardiovasc Prev Rehabil | 14(3) | 441-447 |
| 30 | Berg, S. K. et al. | DenHeart: Differences in physical and mental health across cardiac diagnoses at hospital discharge | 2017 | Journal of Psychosomatic Research | 94 | 1-9 |
| 31 | Biering, K. et al. | Patient-reported health as a prognostic factor for adverse events following percutaneous coronary intervention | 2014 | Clinical Epidemiology | 6 | 61-70 |
| 32 | Björkenheim, A. et al. | Assessment of Atrial Fibrillation-Specific Symptoms Before and 2 Years After Atrial Fibrillation Ablation | 2017 | JACC. Clinical electrophysiology | 3(10) | 1168-1176 |
| 33 | Björkenheim, A. et al. | Patient-Reported Outcomes in Relation to Continuously Monitored Rhythm Before and During 2 Years After Atrial Fibrillation Ablation Using a Disease-Specific and a Generic Instrument | 2018 | Journal of the American Heart Association | 7(5) | e008362 |
| 34 | Blokzijl, F. et al. | The impact of surgical aortic valve replacement on quality of life—a multicenter study | 2021 | The Journal of Thoracic and Cardiovascular Surgery | 161(4) | 1204-1210 |
| 35 | Blumenthal, D. M. et al. | Patient-Reported Outcomes in Cardiology: A Comparison of Two Programs to Assess Angina Burden in Coronary Artery Disease | 2018 | Circ Cardiovasc Qual Outcomes | 11(11) | e004794 |
| 36 | Blumer, V. et al. | Effect of patient-centered transitional care services on patient-reported outcomes in heart failure: sex-specific analysis of the PACT-HF randomized controlled trial | 2021 | European Journal of Heart Failure | 23 | 1488-1498 |
| 37 | Blumer, V. et al. | Sex Differences in Clinical Course and Patient-Reported Outcomes Among Patients Hospitalized for Heart Failure | 2021 | JACC: Heart Failure | 9(5) | 336-345 |
| 38 | Booth, D. et al. | The cost-effectiveness of dapagliflozin in heart failure with preserved or mildly reduced ejection fraction: A European health-economic analysis of the DELIVER trial | 2023 | European Journal of Heart Failure | 25 | 1386-1395 |
| 39 | Borregaard, B. et al. | Sociodemographic, Clinical and Patient-Reported Outcomes and Readmission After Heart Valve Surgery | 2018 | The Journal of heart valve disease | 27(1) | 78-86 |
| 40 | Bouabdallaoui, N. et al. | Beneficial effects of ivabradine in patients with heart failure, low ejection fraction, and heart rate above 77 b.p.m. | 2019 | ESC Heart Failure | 6 | 1199-1207 |
| 41 | Brosved, M. et al. | Effects of Cardiac Rehabilitation on Physical Fitness, Physical Function, and Self-reported Outcomes in Patients ≥80 yr | 2022 | Journal of cardiopulmonary rehabilitation and prevention | 42(5) | 331-337 |
| 42 | Burch, A. E. et al. | Increased Quality of Life Among Newly Diagnosed Patients With Heart Failure With Reduced Ejection Fraction in the Months After Initiation of Guideline-Directed Medical Therapy and Wearable Cardioverter Defibrillator Prescription | 2021 | Journal of Cardiovascular Nursing | 36(6) | 589-594 |
| 43 | Butler, J. et al. | Minimal clinically important difference in quality of life scores for patients with heart failure and reduced ejection fraction | 2020 | European Journal of Heart Failure | 22 | 999-1005 |
| 44 | Butler, J. et al. | Empagliflozin and health-related quality of life outcomes in patients with heart failure with reduced ejection fraction: the EMPEROR-Reduced trial | 2021 | European Heart Journal | 42 | 1203-1212 |
| 45 | Butler, J. et al. | Minimally Clinically Important Difference in Health Status Scores in Patients With HFrEF vs HFpEF | 2022 | JACC: Heart Failure | 10(9) | 651-661 |
| 46 | Butler, J. et al. | Defining changes in physical limitation from the patient perspective: insights from the VITALITY-HFpEF randomized trial | 2022 | European Journal of Heart Failure | 24 | 843-850 |
| 47 | Bäck, M. et al. | Important aspects in relation to patients' attendance at exercise-based cardiac rehabilitation – facilitators, barriers and physiotherapist’s role: a qualitative study | 2017 | BMC Cardiovascular Disorders | 17(1) | 77 |
| 48 | Bäck, M. et al. | Perceptions of Kinesiophobia in Relation to Physical Activity and Exercise After Myocardial Infarction: A Qualitative Study | 2020 | Physical Therapy | 100(12) | 2210-2119 |
| 49 | Bårdsgjerde, E. K. et al. | Patients' narratives of their patient participation in the myocardial infarction pathway | 2019 | Journal of advanced nursing | 75(5) | 1063-1073 |
| 50 | Cakmak, A. S. et al. | Passively Captured Interpersonal Social Interactions and Motion From Smartphones for Predicting Decompensation in Heart Failure: Observational Cohort Study | 2022 | JMIR formative research | 6(8) | e36972 |
| 51 | Campbell, R. T. et al. | Which patients with heart failure should receive specialist palliative care? | 2018 | European Journal of Heart Failure | 20 | 1338-1347 |
| 52 | Candelaria, D. et al. | Validation of the PROMIS-29v2 Health-Related Quality-of-Life Quality-of-Life Questionnaire in Patients With Coronary Heart Disease Participating in Remote Cardiac Rehabilitation | 2022 | Journal of cardiopulmonary rehabilitation and prevention | 42(4) | 246-251 |
| 53 | Candlish, P. et al. | Elderly patients with heart failure: a study of satisfaction with care and quality of life | 1998 | International Journal for Quality in Health Care | 10(2) | 141-146 |
| 54 | Carney, R. et al. | Why people experiencing acute myocardial infarction delay seeking medical assistance | 2002 | European Journal of Cardiovascular Nursing | 1(4) | 237-242 |
| 55 | Chan, P. S. et al. | Development and Validation of a Short Version of the Seattle Angina Questionnaire | 2014 | Circ Cardiovasc Qual Outcomes | 7(5) | 640-647 |
| 56 | Chang, S. et al. | Composite outcome measures in a pragmatic clinical trial of chronic heart failure management: A comparative assessment | 2015 | International Journal of Cardiology | 185 | 62-68 |
| 57 | Charitakis, E. et al. | Factors Predicting Arrhythmia-Related Symptoms and Health-Related Quality of Life in Patients Referred for  Radiofrequency Ablation of Atrial Fibrillation | 2017 | JACC: Clinical Electrophysiology | 3(5) | 494-502 |
| 58 | Chavanon, M. L. et al. | Regional differences in health-related quality of life in elderly heart failure patients: results from the CIBIS-ELD trial | 2017 | Clinical research in cardiology : official journal of the German Cardiac Society | 106(8) | 645-655 |
| 59 | Christensen, A. V. et al. | Increased risk of mortality and readmission associated with lower SF-12 scores in cardiac patients: Results from the national DenHeart study | 2020 | European Journal of Cardiovascular Nursing | 19(4) | 330-338 |
| 60 | Christensen, A. V. et al. | Significantly increased risk of all-cause mortality among cardiac patients feeling lonely | 2020 | Heart (British Cardiac Society) | 106(2) | 140-146 |
| 61 | Christensen, A. V. et al. | Educational inequality in patient-reported outcomes but not mortality among cardiac patients: Results from the national DenHeart survey with register follow-up | 2020 | Scandinavian Journal of Public Health | 48 | 781-790 |
| 62 | Cole, J. A. et al. | Using postal questionnaires to evaluate physical activity and diet behaviour change: case study exploring implications of valid responder characteristics in interpreting intervention outcomes | 2014 | BMC Research Notes | 7 | 725 |
| 63 | Coles, T. M. et al. | Investigating gender-based differential item functioning on the Kansas City Cardiomyopathy Questionnaire (KCCQ) using qualitative content analysis | 2023 | Quality of Life Research | 32(3) | 841-852 |
| 64 | Collet, C. et al. | Differential Improvement in Angina and Health-Related Quality of Life After PCI in Focal and Diffuse Coronary Artery Disease | 2022 | JACC: Cardiovascular Interventions | 15(24) | 2506-2518 |
| 65 | Cosiano, M. F. et al. | Comparing New York Heart Association Class and Patient-Reported Outcomes Among Patients Hospitalized for Heart Failure | 2023 | Circulation. Heart failure | 16(1) | e010107 |
| 66 | Coyne, K. S. et al. | Development and Validation of the AFImpact: An Atrial Fibrillation-Specific Measure of Patient-Reported Health-Related Quality of Life | 2017 | Value in Health | 20(10) | 1355-1361 |
| 67 | Crane, P. B. et al. | Beta-blocker medication usage in older women after myocardial infarction | 2006 | Journal of the American Academy of Nurse Practitioners | 18(10) | 463-470 |
| 68 | Dauerman, H. L. et al. | Durability and Clinical Outcomes of Transcatheter Aortic Valve Replacement for Failed Surgical Bioprostheses | 2019 | Circulation: Cardiovascular Interventions | 12 | e008155 |
| 69 | Daw, P. et al. | A pragmatic effectiveness-implementation study comparing trial evidence with routinely collected outcome data for patients receiving the REACH-HF home-based cardiac rehabilitation programme | 2022 | BMC Cardiovascular Disorders | 22 | 270 |
| 70 | De Smedt, D. et al. | The use of HeartQoL in patients with coronary heart disease: Association with risk factors and European reference values. The EUROASPIRE IV study of the European Society of Cardiology | 2016 | European Journal of Preventive Cardiology | 23(11) | 1174-1186 |
| 71 | De Vos, C. et al. | Participating or not in a cardiac rehabilitation programme: factors influencing a patient’s decision | 2013 | European Journal of Preventive Cardiology | 20(2) | 341-348 |
| 72 | Dehghanzadeh, S. et al. | Living with cardiac resynchronization therapy: Challenges for people with heart failure | 2017 | Nursing and Health Sciences | 19 | 112-118 |
| 73 | Desai, M. Y. et al. | Myosin Inhibition in Patients With Obstructive Hypertrophic Cardiomyopathy Referred for Septal Reduction Therapy | 2022 | Journal of the American College of Cardiology | 80(2) | 95-108 |
| 74 | Ding, Q. et al. | Association of Diabetes Mellitus With Health Status Outcomes in Young Women and Men After acute Myocardial Infarction: Results From the VIRGO Study | 2019 | Journal of the American Heart Association | 8(17) | e010988 |
| 75 | Dong, N. et al. | Relationship Between Patient-Reported Hospital Experience and 30-Day Mortality and Readmission Rates for Acute Myocardial Infarction, Heart Failure, and Pneumonia | 2019 | Journal of general internal medicine | 34(4) | 526-528 |
| 76 | Dreyer, R. P. et al. | Quantifying clinical change: discrepancies between patients' and providers’ perspectives | 2016 | Qual Life Res | 25(9) | 2213-2220 |
| 77 | Dreyer, R. P. et al. | Sex differences in health outcomes at one year following acute myocardial infarction: A report  from the China Patient-Centered Evaluative Assessment of Cardiac Events prospective acute myocardial  infarction study | 2019 | European Heart Journal: Acute Cardiovascular Care | 8(3) | 273-282 |
| 78 | Eldadah, Z. A. et al. | Same-day discharge following catheter ablation and venous closure with VASCADE MVP: A postmarket registry | 2023 | Journal of cardiovascular electrophysiology | 34(2) | 348-355 |
| 79 | Evans, J. M. et al. | Quality of life benefits from arrhythmia ablation: A longitudinal study using the C-CAP questionnaire and EQ5D | 2019 | Pacing and clinical electrophysiology: PACE | 42(6) | 705-711 |
| 80 | Fan, X. et al. | Psychometric testing of the Duke Activity Status Index in patients with heart failure | 2015 | European Journal of Cardiovascular Nursing | 14(3) | 214-221 |
| 81 | Farwati, M. et al. | Super and Nonresponders to Catheter Ablation for Atrial Fibrillation | 2021 | Circulation. Arrhythmia and electrophysiology | 14(8) | e009938 |
| 82 | Farwati, M. et al. | Impact of redo ablation for atrial fibrillation on patient-reported outcomes and quality of life | 2023 | Journal of cardiovascular electrophysiology | 34(1) | 54-61 |
| 83 | Farwati, M. et al. | Cryoballoon pulmonary vein isolation versus radiofrequency ablation of the pulmonary veins and left atrial posterior wall: Patient-reported outcomes | 2024 | Pacing and clinical electrophysiology: PACE | 47(5) | 595-602 |
| 84 | Faxén, U. L. et al. | Patient reported outcome in HFpEF: Sex-specific differences in quality of life and association with outcome | 2018 | International Journal of Cardiology | 267 | 128-132 |
| 85 | Felker, G. M. et al. | Effects of Omecamtiv Mecarbil on Symptoms and Health-Related Quality of Life in Patients With Chronic Heart Failure | 2020 | Circulation. Heart failure | 13(12) | e007814 |
| 86 | Filippatos, G. et al. | Patient-reported outcomes in the SOluble guanylate Cyclase stimulatoR in heArT failurE patientS with PRESERVED ejection fraction (SOCRATES-PRESERVED) study | 2017 | European Journal of Heart Failure | 19 | 782-791 |
| 87 | Flint, K. M. et al. | Variation in clinical and patient-reported outcomes among complex heart failure with preserved  ejection fraction phenotypes | 2020 | ESC Heart Failure | 7 | 811-824 |
| 88 | Flynn, K. E. et al. | Relationships Between Patient-Reported Outcome Measures and Clinical Measures in Outpatients With Heart Failure | 2009 | American Heart Journal | 158(4 Suppl) | S64-S71 |
| 89 | Flynn, K. E. et al. | Reliability and construct validity of PROMIS® Measures for Patients With Heart Failure Who Undergo Heart Transplant | 2015 | Qual Life Res | 24(11) | 2591-2599 |
| 90 | Ford, T. J. et al. | 1-Year Outcomes of Angina Management Guided by Invasive Coronary Function Testing (CorMicA) | 2020 | JACC: Cardiovascular Interventions | 13(1) | 33-45 |
| 91 | Forsyth, P. et al. | Patient-reported barriers to medication adherence in heart failure in Scotland | 2019 | International Journal of Pharmacy Practice | 27 | 443-450 |
| 92 | Frantzen, A. T. et al. | Frailty Status and Patient-Reported Outcomes in Octogenarians Following Transcatheter or Surgical Aortic Valve Replacement | 2021 | Heart, Lung and Circulation | 30 | 1221-1231 |
| 93 | Freedland, K. E. et al. | Use of the PROMIS Depression Scale and the Beck Depression Inventory in Patients with Heart Failure | 2019 | Health Psychol | 38(5) | 369-375 |
| 94 | Freedland, K. E. et al. | Treatment of depression and inadequate self-care in patients with heart failure: One-year outcomes of a randomized controlled trial | 2023 | General Hospital Psychiatry | 84 | 82-88 |
| 95 | Gabilondo, M. et al. | Quality of life in patients with nonvalvular atrial fibrillation treated with oral anticoagulants | 2021 | Hematology | 26(1) | 277-283 |
| 96 | Gisi, B. et al. | The unmeasured burden: Contribution of depression and psychological stress to patient-reported outcomes in atrial fibrillation | 2020 | International Journal of Cardiology | 302 | 75-80 |
| 97 | Gleason, K. T. et al. | Association of sex, age and education level with patient reported outcomes in atrial fibrillation | 2019 | BMC Cardiovascular Disorders | 19(1) | 85 |
| 98 | Gleason, K. T. et al. | Association of sex and atrial fibrillation therapies with patient-reported outcomes | 2019 | Heart (British Cardiac Society) | 105(21) | 1642-1648 |
| 99 | Glickman, S. W. et al. | Patient satisfaction and its relationship with clinical quality and Inpatient Mortality in Acute Myocardial Infarction | 2010 | Circulation. Cardiovascular quality and outcomes | 3(2) | 188-195 |
| 100 | Golbus, J. R. et al. | Association Between Wearable Device Measured Activity and Patient-Reported Outcomes for Heart Failure | 2023 | JACC: Heart Failure | 11(11) | 1521-1530 |
| 101 | Gopinathannair, R. et al. | Longitudinal changes in quality of life following ICD implant and the impact of age, gender, and ICD shocks: observations from the INTRINSIC RV trial | 2017 | J Interv Card Electrophysiol | 48(3) | 291-298 |
| 102 | Gott, M. et al. | Patient views of social service provision for older people with advanced heart failure | 2007 | Health & social care in the community | 15(4) | 333-342 |
| 103 | Goyal, P. et al. | Patient-Reported Barriers and Facilitators to Deprescribing Cardiovascular Medications | 2020 | Drugs & aging | 37(2) | 125-135 |
| 104 | Grady, K. L. et al. | Novel measures to assess ventricular assist device patient-reported outcomes: Findings from the MCS A-QOL study | 2024 | The Journal of Heart and Lung Transplantation | 43(1) | 36-50 |
| 105 | Greene, S. J. et al. | Comparison of New York Heart Association Class and Patient-Reported Outcomes for Heart Failure With Reduced Ejection Fraction | 2021 | JAMA Cardiology | 6(5) | 522-531 |
| 106 | Greene, S. J. et al. | Comparative Effectiveness of Dosing of Medical Therapy for Heart Failure: From the CHAMP-HF Registry | 2022 | Journal of Cardiac Failure | 28(3) | 370-383 |
| 107 | Greene, S. J. et al. | Effect of Torsemide Versus Furosemide on Symptoms and Quality of Life Among Patients Hospitalized for Heart Failure: The TRANSFORM-HF Randomized Clinical Trial | 2023 | Circulation | 148(2) | 124-134 |
| 108 | Groene, O. et al. | Patient Experience Shows Little Relationship with Hospital Quality Management Strategies | 2015 | PloS one | 10(7) | e0131805 |
| 109 | Guidotti, E. et al. | A longitudinal assessment of chronic care pathways in real-life: self-care and outcomes of chronic heart failure patients in Tuscany | 2022 | BMC Health Services Research | 22 | 1146 |
| 110 | Guimarães, W. V. N. et al. | Seattle Angina Pectoris Questionnaire and Canadian Cardiovascular Society Angina Categories in the Assessment of Total Coronary Atherosclerotic Burden | 2021 | The American Journal of Cardiology | 152 | 43-48 |
| 111 | Gundersen, G. H. et al. | Patient-reported outcomes and associations with pleural effusion in outpatients with heart failure: an observational cohort study | 2017 | BMJ Open | 7(3) | e013734 |
| 112 | Guo, X. et al. | Patient reported outcomes and quality of life in Chinese patients with implantable cardioverter defibrillators | 2021 | Heart & Lung | 50 | 153-158 |
| 113 | Gustafson, D. H. et al. | Increasing understanding of patient needs during and after hospitalization | 2001 | Journal on Quality Improvement | 27(2) | 81-92 |
| 114 | Güder, G. et al. | Nurse-coordinated collaborative disease management improves the quality of guideline-recommended heart failure therapy, patient-reported outcomes, and left  ventricular remodelling | 2015 | European Journal of Heart Failure | 17 | 442-452 |
| 115 | Habibović, M. et al. | Gender disparities in anxiety and quality of life in patients with an implantable cardioverter-defibrillator | 2011 | Europace | 13 | 1723-1730 |
| 116 | Habibović, M. et al. | Poor health status and distress in cardiac patients: the role of device therapy vs. underlying heart disease | 2013 | Europace | 15 | 355-361 |
| 117 | Habibović, M. et al. | Web-based distress management for implantable cardioverter defibrillator patients: A randomized controlled trial | 2017 | Health Psychology | 36(4) | 392-401 |
| 118 | Han, Q. et al. | A nomogram based on a patient-reported outcomes measure: predicting the risk of readmission for patients with chronic heart failure | 2020 | Health and Quality of Life Outcomes | 18(1) | 290 |
| 119 | Hanon, O. et al. | Patient-Reported Treatment Satisfaction with Rivaroxaban for Stroke Prevention in Atrial Fibrillation. A French Observational Study, the SAFARI Study | 2016 | PloS one | 11(12) | e0166218 |
| 120 | Harzand, A. et al. | Effects of a patient-centered digital health intervention in patients referred to cardiac rehabilitation: the Smart HEART clinical trial | 2023 | BMC Cardiovascular Disorders | 23 | 453 |
| 121 | Hashimoto, K. et al. | Burden of Mild (<13 g/dl) Anemia in Patients With Atrial Fibrillation (A Report from a Multicenter Registry With Patient-Reported Outcomes) | 2021 | The American Journal of Cardiology | 157 | 48-55 |
| 122 | Hashimoto, S. et al. | Confidence in self-care after heart failure hospitalization | 2023 | Journal of Cardiology | 81 | 42-48 |
| 123 | Hattler, B. et al. | Clinical and Angiographic Predictors of Patient-Reported Angina 1 Year After Coronary Artery Bypass Graft Surgery | 2019 | Circulation. Cardiovascular Quality and Outcomes | 12 | e005119 |
| 124 | Haugaa, K. H. et al. | Patients' knowledge and attitudes regarding living with implantable electronic devices: results of a multicentre, multinational patient survey conducted by the European Heart Rhythm Association | 2018 | Europace | 20(2) | 386-391 |
| 125 | Hayat, A. et al. | Direct oral anticoagulants: patient reported adherence and minor bleedings | 2023 | Journal of Thrombosis and Thrombolysis | 56 | 55-64 |
| 126 | Heimburg, K. et al. | Self-reported limitations in physical function are common 6 months after out-of-hospital cardiac arrest | 2022 | Resuscitation Plus | 11 | 100275 |
| 127 | Heiskanen, J. et al. | Targeted identification of adverse events in coronary artery disease patients based on patient-reported outcomes | 2017 | Journal of Comparative Effectiveness Research | 6(7) | 583-589 |
| 128 | Hengstenberg, C. et al. | Treatment Satisfaction and Convenience for Patients With Atrial Fibrillation on Edoxaban or Vitamin K Antagonists After Transcatheter Aortic Valve Replacement: A Post Hoc Analysis from the ENVISAGE-TAVI AF Trial | 2023 | The American journal of cardiology | 209 | 212-219 |
| 129 | Herlitz, J. et al. | Quality of life 15 years after coronary artery bypass grafting | 2009 | Coronary Artery Disease | 20(6) | 363-369 |
| 130 | Herman, D. et al. | Deactivation of implantable cardioverter-defibrillators: results of patient surveys | 2013 | Europace | 15 | 963-969 |
| 131 | Hernández Madrid, A. et al. | Differences in attitude, education, and knowledge about oral anticoagulation therapy among patients with atrial fibrillation in Europe: result of a self-assessment patient survey conducted by the European Heart Rhythm Association | 2016 | Europace | 18 | 463-467 |
| 132 | Herring, L. Y. et al. | Physical Activity after Cardiac EventS (PACES): a group education programme with subsequent text message support designed to increase physical activity in individuals with diagnosed coronary heart disease: a randomised | 2021 | Trials | 19(1) | 537 |
| 133 | Hilt, A. D. et al. | Perspectives of Patients and Professionals on Information and Education After Myocardial Infarction With Insight for Mixed Reality Implementation: Cross-Sectional Interview Study | 2020 | JMIR Human Factors | 7(2) | e17147 |
| 134 | Hirai, T. et al. | Impact of subintimal plaque modification procedures on health status after unsuccessful chronic total occlusion angioplasty | 2018 | Catheter Cardiovasc Interv | 91(6) | 1035-1042 |
| 135 | Hoegh, V. et al. | Association between the diagnosis of atrial fibrillation and aspects of health status: a Danish cross-sectional study | 2016 | Scandinavian journal of caring sciences | 30(3) | 507-517 |
| 136 | Hong, C. et al. | Acceptability, Preferred Medium, and Components of Nurse-Led Cardiac Telerehabilitation: A Cross-Sectional Study | 2024 | Clinical Nursing Research | 33(2-3) | 146-156 |
| 137 | Hoogwegt, M. T. et al. | Exercise mediates the association between positive affect and 5-Year Mortality in Patients With Ischemic Heart Disease | 2013 | Circulation. Cardiovascular quality and outcomes | 6(5) | 559-566 |
| 138 | Huang, W. et al. | Patient-reported outcomes in heart failure with preserved vs. reduced ejection fraction: focus on physical independence | 2020 | ESC heart failure | 7(5) | 2051-2062 |
| 139 | Humphries, B. et al. | Patient-Reported Outcomes and Patient-Reported Experience of Patients With Atrial Fibrillation in the IMPACT-AF Clinical Trial | 2021 | Journal of the American Heart Association | 10 | e019783 |
| 140 | Hussein, A. A. et al. | New Model of Automated Patient-Reported Outcomes Applied in Atrial Fibrillation | 2019 | Circulation. Arrythmia and Electrophysiology | 12(3) | e006986 |
| 141 | Hutchings, H. A. et al. | Can we collect health-related quality of life information from anticoagulated atrial fibrillation participants who have recently experienced a bleed? An observational feasibility study in primary and secondary care in Wales  and through a UK online forum | 2023 | BMJ Open | 13 | e075335 |
| 142 | Hwang, B. et al. | Effects of an Educational Intervention on Heart Failure  Knowledge, Self-Care Behaviors, and Health-Related Quality of Life of Patients with Heart Failure: Exploring the Role of Depression | 2020 | Patient education and counseling | 103(6) | 1201-1208 |
| 143 | Hwang, B. et al. | Effects of educational intervention on mortality and patient-reported outcomes in individuals with heart failure: A randomized controlled trial | 2022 | Patient Education and Counseling | 105 | 2740-2746 |
| 144 | Hwang, R. et al. | Home-based telerehabilitation is not inferior to a centre-based program in patients with chronic heart failure: a randomised trial | 2017 | Journal of Physiotherapy | 63 | 101-107 |
| 145 | Höfer, S. et al. | The MacNew Heart Disease Health-Related Quality of Life Questionnaire in Patients with Angina and Patients with Ischemic Heart Failure | 2012 | Value in Health | 15(1) | 143-150 |
| 146 | Ikemura, N. et al. | Novel Approach for Visualizing Multiple Domains of Quality of Life Scales | 2019 | Circulation. Cardiovascular quality and outcomes | 12 | e005573 |
| 147 | Ikemura, N. et al. | Cohort profile: patient characteristics and quality-of-life measurements for newly-referred patients with atrial fibrillation—Keio interhospital Cardiovascular Studies-atrial fibrillation (KiCS-AF) | 2019 | BMJ Open | 9 | e032746 |
| 148 | Ikemura, N. et al. | Baseline and Postprocedural Health Status Outcomes in Contemporary Patients With Atrial Fibrillation Who Underwent Catheter Ablation: A Report from the Japanese Outpatient Registry | 2021 | Journal of the American Heart Association | 10 | e019983 |
| 149 | Ikemura, N. et al. | Physician Estimates and Patient-Reported Health Status in Atrial Fibrillation | 2024 | JAMA network open | 7(2) | e2356693 |
| 150 | Ingadottir, B. et al. | Patients are expecting to learn more: A longitudinal study of patients with heart failure undergoing device implantation | 2020 | Patient Education and Counseling | 103(7) | 1382-1389 |
| 151 | Jaarsma, T. et al. | Changes over time in patient-reported outcomes in patients with heart failure | 2024 | ESC Heart Failure | 11 | 811-818 |
| 152 | Jabbar, A. et al. | Effect of Levothyroxine on Left Ventricular Ejection Fraction in Patients With Subclinical Hypothyroidism and Acute Myocardial Infarction | 2020 | JAMA | 324(3) | 249-258 |
| 153 | Jackson, E. A. et al. | Perceived Quality of Care and Lifestyle Counseling Among Patients With Heart Disease | 2010 | Clinical Cardiology | 33(12) | 765-769 |
| 154 | Jankowska-Polańska, B. et al. | Patient-Reported Compliance in older age patients with chronic heart failure | 2020 | PloS one | 15(4) | e0231076 |
| 155 | Jarmoszewicz, K. et al. | Predictors of patient-reported health following cardiac surgery | 2021 | The Journal of Cardiovascular Surgery | 62(3) | 278-285 |
| 156 | Jenkinson, C. et al. | The coronary heart disease in-patient experience questionnaire (I-PEQ (CHD)): Results from the survey of National Health Service patients | 2002 | Quality of Life Research | 11 | 721-727 |
| 157 | Jenkinson, C. et al. | Factors relating to patients' reports about hospital care for coronary heart disease in England | 2003 | Journal of Health Services Research & Policy | 8(2) | 83-86 |
| 158 | Jennings, C. S. et al. | ASPIRE-3-PREVENT: a cross-sectional survey of preventive care after a coronary event across the UK | 2020 | Open Heart | 7(1) | e001196 |
| 159 | Jin, H. et al. | Age-related differences in factors associated with the underuse of recommended medications in acute coronary syndrome patients at least one year after hospital discharge | 2014 | BMC Cardiovascular Disorders | 14 | 127 |
| 160 | Johnson, B. M. et al. | Atrial Fibrillation Ablation in Young Adults: Measuring Quality of Life using Patient Reported Outcomes over 5 Years | 2023 | Circulation. Arrhythmia and electrophysiology | 16(6) | e011565 |
| 161 | Jonsson, H. et al. | Is frailty associated with long-term survival, neurological function and patient-reported outcomes after in-hospital cardiac arrest? – A Swedish cohort study | 2022 | Resuscitation | 179 | 233-242 |
| 162 | Jourdain, P. et al. | Perceptions and satisfaction of patients with chronic heart failure when using a remote monitoring web application named Satelia® Cardio | 2023 | Annales de cardiologie et d’angeiologie | 72(3) | 101606 |
| 163 | Kandzari, D. E. et al. | Procedural, clinical, and health status outcomes in chronic total coronary occlusion revascularization: Results from the PERSPECTIVE study | 2020 | Catheter Cardiovasc Interv | 96(3) | 567-576 |
| 164 | Kane, P. M. et al. | Feasibility and acceptability of a patient-reported outcome intervention in chronic heart failure | 2017 | BMJ Support Palliat Care | 7(4) | 470-479 |
| 165 | Kane, P. M. et al. | Understanding how a palliative-specific patient-reported outcome intervention works to facilitate patient-centred care in advanced heart failure: A qualitative study | 2018 | Palliative Medicine | 32(1) | 143-155 |
| 166 | Kaufman, B. G. et al. | Disease understanding in patients newly diagnosed with atrial fibrillation | 2018 | Heart (British Cardiac Society) | 104(6) | 494-501 |
| 167 | Kaul, P. et al. | Patient-Reported Frailty and Functional Status in Heart Failure With Preserved Ejection Fraction | 2023 | JACC: Heart Failure | 11(4) | 392-403 |
| 168 | Kayaniyil, S. et al. | Degree and correlates of patient trust in their cardiologist | 2009 | Journal of evaluation in clinical practice | 15(4) | 634-640 |
| 169 | Kedžo, J. et al. | Neurologic Biomarkers, Neuroimaging, and Cognitive Function in Persistent Atrial Fibrillation: A Cross-Sectional Study | 2023 | International Journal of Molecular Sciences | 24 | 2902 |
| 170 | Kelly-Blake, K. et al. | Refining a brief decision aid in stable CAD: cognitive interviews | 2014 | BMC Medical Informatics & Decision Making | 14 | 10 |
| 171 | Kemp, I. et al. | A comparison of angina symptoms reported by clinicians and patients, pre and post revascularization: Insights from the Stent or Surgery Trial | 2019 | International Journal of Cardiology | 293 | 25-31 |
| 172 | Kemp, K. A. et al. | Sex Differences in the Care Experiences of Patients Hospitalized Due to Ischemic Heart Disease in Alberta Canada | 2021 | CJC open | 3(12 Suppl) | S36-S43 |
| 173 | Ketilsdottir, A. et al. | Self-reported health and quality of life outcomes of heart failure patients in the aftermath of a national economic crisis: a cross-sectional study | 2019 | ESC Heart Failure | 6 | 111-121 |
| 174 | Khajavi, A. et al. | Chronic heart failure health-related quality of life questionnaire (CHFQOLQ-20): development and psychometric properties | 2023 | BMC Cardiovascular | 23 | 165 |
| 175 | Khalili, H. et al. | Quality of Life Outcomes After Transcatheter Aortic Valve Replacement in Nonagenarians | 2020 | Journal of Invasive Cardiology | 32(10) | 375-379 |
| 176 | Khan, M. S. et al. | Effect of Carillon Mitral Contour System on patient-reported outcomes in functional mitral regurgitation: an individual participant data meta-analysis | 2021 | ESC Heart Failure | 8 | 1885-1891 |
| 177 | Kim, I. C. et al. | Physician adherence and patient-reported outcomes in heart failure | 2022 | Scientific reports | 12(1) | 7730 |
| 178 | Kitakata, H. et al. | Patient confidence regarding secondary lifestyle modification and knowledge of ‘heart attack’ symptoms following percutaneous revascularisation in Japan:  a cross-sectional study | 2018 | BMJ Open | 8 | e019119 |
| 179 | Kitakata, H. et al. | Social Isolation and Implementation of Advanced Care Planning Among Hospitalized Patients With Heart Failure | 2022 | Journal of the American Heart Association | 11 | e026645 |
| 180 | Kjellsdotter, A. et al. | Associations Between Sleep and Personality Factors Among Patients Living With Coronary Artery Disease | 2020 | Journal of Cardiovascular Nursing | 35(6) | 568-575 |
| 181 | Klavebäck, S. et al. | Management of modifiable risk factors and comorbidities in atrial fibrillation: suggestions for improvement from a patient perspective | 2024 | European Journal of Cardiovascular Nursing | 23 | 169-175 |
| 182 | Kofi Okoh, A. et al. | Association of Change in Patient-Reported Health Status after Transcatheter Aortic Valve Replacement, and Postoperative Outcomes | 2017 | The Journal of heart valve disease | 26(5) | 493-501 |
| 183 | Kondo, T. et al. | Use of Win Statistics to Analyze Outcomes in the DAPA-HF and DELIVER Trial | 2023 | NEJM Evid | 2(11) | EVIDoa2300042 |
| 184 | Koo, B. K. et al. | Fractional Flow Reserve or Intravascular Ultrasonography to Guide PCI | 2022 | The New England Journal of Medicine | 387(9) | 779-789 |
| 185 | Koretsune, Y. et al. | Patient-reported treatment satisfaction with rivaroxaban in Japanese non-valvular atrial fibrillation patients: an observational study | 2018 | Current medical research and opinion | 34(12) | 2157-2164 |
| 186 | Kowalyk, K. M. et al. | Measuring continuity of care for cardiac patients: Development of a patient self-report questionnaire | 2004 | The Canadian Journal of Cardiology | 20(2) | 205-212 |
| 187 | Kraai, I. H. et al. | Perception of impairments by patients with heart failure. | 2016 | European journal of cardiovascular nursing | 15(2) | 178-185 |
| 188 | Kraai, I. et al. | The value of telemonitoring and ICT-guided disease management in heart failure: Results from the IN TOUCH study | 2016 | International journal of medical informatics | 85(1) | 53-60 |
| 189 | Krack, G. et al. | Determinants of adherence and effects on health-related quality of life after myocardial infarction: a prospective cohort study | 2018 | BMC Geriatrics | 18 | 136 |
| 190 | Kristensen, M. S. et al. | Validating the HeartQoL questionnaire in patients with atrial fibrillation | 2016 | European Journal of Preventive Cardiology | 23(14) | 1496-1503 |
| 191 | Kugler, C. et al. | Sexual activity in patients with left ventricular assist device and their partners: impact of the device on quality of life, anxiety and depression | 2018 | European Journal of Cardio-Thoracic Surgery | 53 | 799-806 |
| 192 | Kwon, J. Y. et al. | Patient-reported outcomes and the identification of subgroups of atrial fbrillation patients: a retrospective cohort study of linked clinical registry and administrative data | 2021 | Quality of Life Research | 30 | 1547-1559 |
| 193 | Kähkönen, O. et al. | Perceived health among percutaneous coronary intervention patients over a six-year follow-up period | 2023 | Journal of Clinical Nursing | 32 | 4816-4826 |
| 194 | König, S. et al. | Patient perspectives on same-day discharge following catheter ablation for atrial fibrillation: results from a patient survey as part of the monocentric FAST AFA trial | 2023 | Europace | 25 | 1-6 |
| 195 | Lagha, E. et al. | Patient Reported Experience Measures (PREMs) in chronic heart failure | 2012 | J R Coll Physicians Edinb | 42(4) | 301-305 |
| 196 | Lainscak, M. & Keber, I. | Patient's view of heart failure: from the understanding to the quality of life | 2003 | European Journal of Cardiovascular Nursing | 2 | 275-281 |
| 197 | Lane, D. A. et al. | Atrial fibrillation patient preferences for oral anticoagulation and stroke knowledge: Results of a conjoint analysis | 2018 | Clinical cardiology | 41(6) | 855-861 |
| 198 | Lappalainen, L. et al. | Patient-reported outcomes in coronary artery disease: the relationship between the standard, disease-specifc  set by the International Consortium for Health Outcomes Measurement (ICHOM) and the generic health-related quality of life instrument 15D | 2021 | Health and quality of life outcomes | 19 | 206 |
| 199 | Larsen, J. M. et al. | The patient perspective on the Riata defibrillator lead advisory: A Danish nationwide study | 2014 | Heart rhythm | 11(12) | 2148-2155 |
| 200 | Larsen, K. K. et al. | Screening for depression in patients with myocardial infarction by general practitioners | 2013 | European Journal of Preventive Cardiology | 20(5) | 800-806 |
| 201 | Lavikainen, P. et al. | Agreement between physician- and patient-reported Canadian cardiovascular society scores among patients undergoing elective coronary angiography-The CATS study | 2023 | PLoS ONE | 18(10) | e0292058 |
| 202 | Lawal, O. A. et al. | Response shift in coronary artery disease | 2024 | Quality of Life Research | 33 | 767-776 |
| 203 | Lee, M. M. Y. et al. | Health-related quality of life in acute heart failure: association between patient-reported symptoms and markers of congestion | 2023 | European Journal of Heart Failure | 25(1) | 54-60 |
| 204 | Lee, W. L. et al. | Test-retest reliability of *HeartQoL* and its comparability to the *MacNew* heart disease health-related quality of life questionnaire | 2016 | Qual Life Res | 25(2) | 531-357 |
| 205 | Leppert, F. et al. | The INFluence of Remote monitoring on Anxiety/depRession, quality of lifE, and Device acceptance in ICD patients: a prospective, randomized, controlled, single‑center trial | 2021 | Clinical Research in Cardiology | 110 | 789-800 |
| 206 | Lilja, G. et al. | Anxiety and depression among out-of-hospital cardiac arrest survivors | 2015 | Resuscitation | 97 | 68-75 |
| 207 | Lind, L. et al. | Old-and With Severe Heart Failure: Telemonitoring by Using Digital Pen Technology in Specialized Homecare: System Description, Implementation, and Early Results | 2016 | Computers, informatics, nursing : CIN | 34(8) | 360-368 |
| 208 | Lu, Y. et al. | Sex Differences in Omega-3 and -6 Fatty Acids and Health Status Among Young Adults With Acute Myocardial Infarction: Results From the VIRGO Study | 2018 | Journal of the American Heart Association | 7(11) | e008189 |
| 209 | Ludt, S. et al. | The challenge of cardiovascular prevention in primary care: implications of a European observational study in 8928 patients at different risk levels | 2014 | European Journal of Preventive Cardiology | 21(2) | 203-213 |
| 210 | Lum, H. D. et al. | Burdensome Physical and Depressive Symptoms Predict Heart Failure - Specific Health Status Over One Year | 2016 | Journal of Pain and Symptom Management | 51(6) | 963-970 |
| 211 | Luo, N. et al. | Multinational and multiethnic variations in health-related quality of life in patients with chronic heart failure | 2017 | American Heart Journal | 191 | 75-81 |
| 212 | Luo, N. et al. | Relationship between changing patient-reported outcomes and subsequent clinical events in patients with chronic heart failure: insights from HF-ACTION | 2019 | European Journal of Heart Failure | 21 | 63-70 |
| 213 | Madigan, E. A. | People with heart failure and home health care resource use and outcomes | 2008 | Journal of clinical nursing | 17(7B) | 253-259 |
| 214 | Mahé, I. et al. | Use and monitoring of vitamin K antagonists in everyday medical practice | 2006 | La Presse Médicale | 35(12) | 1797-1803 |
| 215 | Malhotra, C. et al. | Financial difficulties and patient-reported outcomes among patients with advanced heart failure | 2021 | Quality of Life Research | 30 | 1379-1387 |
| 216 | Manemann, S. M. et al. | Perceived Social Isolation and Outcomes in Patients With Heart Failure | 2018 | Journal of the American Heart Association | 7(11) | e008069 |
| 217 | Mangal, S. et al. | Perceptions of patient-reported outcome data access and sharing among patients with heart failure: ethical implications for research | 2024 | European Journal of Cardiovascular Nursing | 23 | 145-151 |
| 218 | Masdjedi, K. et al. | A case-vignette based assessment of patient's perspective on coronary revascularization strategies, the OPINION study | 2018 | Journal of Cardiology | 72 | 149-154 |
| 219 | Mastenbroek, M. H. et al. | Relationship Between Reverse Remodeling and Cardiopulmonary Exercise Capacity in Heart Failure Patients Undergoing Cardiac Resynchronization Therapy | 2016 | Journal of Cardiac Failure | 22(5) | 385-394 |
| 220 | Mathisen, L. et al. | Patient-Reported Outcome After Randomization to  On-Pump Versus Off-Pump Coronary Artery Surgery | 2005 | The Annals of Thoracic Surgery | 79(5) | 1584-1589 |
| 221 | McEwan, P. et al. | Cost-effectiveness of dapagliflozin as a treatment for heart failure with reduced ejection fraction: a multinational health-economic analysis of DAPA-HF | 2020 | European Journal of Heart Failure | 22 | 2147-2156 |
| 222 | McKinley, S. et al. | International comparison of factors associated with delay in presentation for AMI treatment | 2004 | European Journal of Cardiovascular Nursing | 3 | 225-230 |
| 223 | Meng, Y. et al. | A Machine Learning Approach to Classifying Self-Reported Health Status in a cohort of Patients with Heart Disease using Activity Tracker Data | 2020 | IEEE journal of biomedical and health informatics | 24(3) | 878-884 |
| 224 | Mentz, R. J. et al. | PredischaRge initiation of Ivabradine in the ManagEment of Heart Failure: Results of the PRIME-HF Trial | 2020 | American Heart Journal | 223 | 98-105 |
| 225 | Mentz, R. J. et al. | PROVIDE-HF primary results: Patient-Reported Outcomes inVestigation following Initiation of Drug therapy with Entresto (sacubitril/valsartan) in heart failure | 2020 | American Heart Journal | 230 | 35-43 |
| 226 | Meterko, M. et al. | Mortality among Patients with Acute Myocardial Infarction: The Influences of Patient-Centered Care and  Evidence-Based Medicine | 2010 | Health Services Research | 45(5 Pt 1) | 1188-1204 |
| 227 | Michelis, K. C. et al. | Discordance Between Severity of Heart Failure as Determined by Patient Report Versus Cardiopulmonary Exercise Testing | 2021 | Journal of the American Heart Association | 10 | e019864 |
| 228 | Middel, B. et al. | Decline in health-related quality of life 6 months after Coronary Artery Bypass Graft Surgery | 2014 | Journal of Cardiovascular Nursing | 29(6) | 544-554 |
| 229 | Miura, K. et al. | Treatment strategies and subsequent changes in the patient-reported quality-of-life among elderly patients with atrial fibrillation | 2020 | American Heart Journal | 222 | 83-92 |
| 230 | Mols, R. E. et al. | Patient-reported outcome is associated with health care costs in patients with ischaemic heart disease and arrythmia | 2023 | European Journal of Cardiovascular Nursing | 22 | 23-32 |
| 231 | Mommersteeg, P. M. et al. | Impaired Health Status, Psychological Distress, and Personality in Women and Men With Nonobstructive Coronary Artery Disease | 2017 | Circ Cardiovasc Qual Outcomes | 10 | e003397 |
| 232 | Mondesir, F. L. et al. | Patient Perspectives on the Completion and Use of Patient-Reported outcome surveys in routine clinical care for heart failure | 2020 | Circ Cardiovasc Qual Outcomes | 13(9) | e007027 |
| 233 | Morgan, D. M. | Effect of incongruence of acute myocardial infarction symptoms on the decision to seek treatment in a rural population | 2005 | Journal of Cardiovascular Nursing | 20(5) | 365-371 |
| 234 | Moser, D. K. et al. | Gender differences in reasons patients delay in seeking treatment for acute myocardial infarction symptoms | 2005 | Patient Education and Counseling | 56 | 45-54 |
| 235 | Munyombwe, T. et al. | Association of multimorbidity and changes in health-related quality of life following myocardial infarction: a UK multicentre longitudinal patient-reported outcomes study | 2021 | BMC Medicine | 19 | 227 |
| 236 | Nagl, M. & Farin, E. | The development of an instrument assessing the relevance of rehabilitation outcomes for patient participation: Retest reliability and descriptive results | 2011 | Die Rehabilitation | 50(6) | 379-389 |
| 237 | Nakano, A. et al. | Patient perception and assessment of admission to acute cardiac care unit | 2008 | European Journal of Cardiovascular Nursing | 7 | 10-15 |
| 238 | Nielsen, M. H. et al. | Return to work and everyday life following out-of-hospital cardiac arrest. Results from the national survey, DenHeart | 2023 | Heart & Lung | 58 | 54-61 |
| 239 | Nieminen, M. S. et al. | Oral levosimendan in patients with severe chronic heart failure—The PERSIST study | 2008 | European Journal of Heart Failure | 10 | 1246-1254 |
| 240 | Nobel, L. et al. | Neighborhood Socioeconomic Status Predicts Health After Hospitalization for Acute Coronary Syndromes: findings from TRACE-CORE (Transitions, Risks, and Actions in Coronary Events – Center for Outcomes Research and Education) | 2017 | Medical Care | 55(12) | 1008-1016 |
| 241 | Nolan, R. P. et al. | Automated E-Counseling for Chronic Heart Failure: CHF-CePPORT Trial | 2021 | Circulation. Heart Failure | 14(1) | e007073 |
| 242 | Nordgren, L. et al. | An exploration of the phenomenon of formal care from the perspective of middle-aged heart failure patients | 2007 | European Journal of Cardiovascular Nursing | 6 | 121-129 |
| 243 | Norekvål, T. M. et al. | Patient-reported outcomes as predictors of 10-year survival in women after acute myocardial infarction | 2010 | Health and Quality of Life Outcomes | 8 | 140 |
| 244 | Norman, J. et al. | Effects of a mindfulness-based intervention on symptoms and signs in chronic heart failure: A feasibility study | 2018 | European Journal of Cardiovascular Nursing | 17(1) | 54-65 |
| 245 | Noureddine, S. | Patterns of responses to cardiac events over time | 2009 | Journal of Cardiovascular Nursing | 24(5) | 390-397 |
| 246 | Noureddine, S. et al. | Delay in seeking health care for acute coronary syndromes in a Lebanese Sample | 2006 | Journal of Transcultural Nursing | 17 | 241-348 |
| 247 | Obamiro, K. O. et al. | Adherence to Oral Anticoagulants in Atrial Fibrillation: An Australian Survey | 2018 | Journal of Cardiovascular Pharmacology and Therapeutics | 23(4) | 337-343 |
| 248 | Ocagli, H. et al. | Monitoring Patients Reported Outcomes after Valve Replacement Using Wearable Devices: Insights on Feasibility and Capability Study: Feasibility Results | 2021 | International journal of environmental research and public health | 18(13) | 7171 |
| 249 | Oinasmaa, S. et al. | Does routinely collected patient-reported outcome data represent the actual case-mix of elective coronary revascularization patients? | 2018 | European heart journal. Quality of care & clinical outcomes | 4(2) | 113-119 |
| 250 | Okunrintemi, V. et al. | Gender Differences in Patient-Reported Outcomes Among Adults With Atherosclerotic Cardiovascular Disease | 2018 | Journal of the American Heart Association | 7(24) | e010498 |
| 251 | Okunrintemi, V. et al. | Association of Depression Risk with Patient Experience, Healthcare Expenditure, and Health Resource Utilization Among Adults with Atherosclerotic Cardiovascular Disease | 2019 | Journal of general internal medicine | 34(11) | 2427-2434 |
| 252 | Olsen, S. J. et al. | Changes in self-reported health and quality of life in octogenarian patients one month after transcatheter aortic valve implantation | 2017 | European Journal of Cardiovascular Nursing | 16(1) | 79-87 |
| 253 | Ono, F. et al. | Impact of care coordination on oral anticoagulant therapy among patients with atrial fibrillation in routine clinical practice in Japan: a prospective, observational study | 2019 | BMC Cardiovascular Disorders | 19 | 235 |
| 254 | Ono, M. et al. | Effect of Patient-Reported Preprocedural Physical and Mental Health on 10-Year Mortality After Percutaneous or Surgical Coronary Revascularization | 2022 | Circulation | 146(17) | 1268-1280 |
| 255 | Ozdemir, S. et al. | A Prospective Cohort Study of Medical Decision-Making Roles and Their Associations with Patient Characteristics and Patient-Reported Outcomes among Patients with Heart Failure | 2023 | Med Decis Making | 43(7-8) | 863-874 |
| 256 | Patrick, M. et al. | Anxiety and depression moderate the relationship between quality of life and self-care in patients with heart failure | 2022 | Geriatric Nursing | 44 | 54-59 |
| 257 | Pavlovic, N. et al. | Identification of Fatigue Subtypes and Their Correlates in Prevalent Heart Failure: A Secondary Analysis of the Atherosclerosis Risk in Communities Study | 2024 | Circulation. Cardiovascular Quality and Outcomes | 17 | e010115 |
| 258 | Pedersen, C. G. et al. | Cardiac rehabilitation: pedagogical education strategies have positive effect on long-term patient-reported outcomes | 2023 | Health Education Research | 38(6) | 597-609 |
| 259 | Pedersen, S. S. et al. | Patients' perspective on deactivation of the implantable cardioverter-Defibrillator Near the End of Life | 2013 | The American journal of cardiology | 111(10) | 1443-1447 |
| 260 | Pennucci, F. et al. | Piloting a web-based systematic collection and reporting of patient-reported outcome measures and patient-reported experience measures in chronic heart failure | 2020 | BMJ open | 10(10) | e037754 |
| 261 | Perino, A. C. et al. | Comparison of Patient-Reported Care Satisfaction, Quality of Warfarin Therapy, and Outcomes of Atrial Fibrillation: Findings From the ORBIT-AF Registry | 2019 | Journal of the American Heart Association | 8(9) | e011205 |
| 262 | Peters, A. E. et al. | Patient-reported and Clinical Outcomes Among Patients Hospitalized for Heart Failure With Reduced Versus Preserved Ejection Fraction | 2022 | Journal of Cardiac Failure | 28(12) | 1652-1660 |
| 263 | Peters, M. et al. | Change in health status in long-term conditions over a one year period: a cohort survey using patient-reported outcome measures | 2014 | Health and Quality of Life Outcomes | 12 | 123 |
| 264 | Pettersen, T. R. et al. | Challenges adhering to a medication regimen following first-time percutaneous coronary intervention: A patient perspective | 2018 | International Journal of Nursing Studies | 88 | 16-24 |
| 265 | Pfaeffli Dale, L. et al. | Acceptability of a mobile health exercise-based cardiac rehabilitation intervention | 2015 | Journal of Cardiopulmonary Rehabilitation and Prevention | 35 | 312-319 |
| 266 | Piña, I. L. et al. | Improvement of Health Status Following Initiation of Sacubitril/Valsartan in Heart Failure and Reduced Ejection Fraction | 2021 | JACC: Heart Failure | 9(1) | 42-51 |
| 267 | Pocock, S. J. et al. | The win ratio method in heart failure trials: lessons learnt from empulse | 2023 | European journal of heart failure | 25(5) | 632-641 |
| 268 | Pokharel, Y. et al. | Association of Serial Kansas City Cardiomyopathy Questionnaire Assessments With Death and Hospitalization in Patients With Heart Failure With Preserved and Reduced Ejection Fraction | 2017 | JAMA Cardiology | 2(12) | 1315-1321 |
| 269 | Potpara, T. S. et al. | Self-reported treatment burden in patients with atrial fibrillation: quantification, major determinants, and implications for integrated holistic management of the arrhythmia | 2020 | Europace | 22 | 1788-1797 |
| 270 | Prescher, S. et al. | Telemedical care: feasibility and perception of the  patients and physicians: a survey-based acceptance  analysis of the Telemedical Interventional Monitoring  in Heart Failure (TIM-HF) trial | 2013 | European Journal of Preventive Cardiology | 20(2 Suppl) | 18-24 |
| 271 | Prescher, S. et al. | Patient reported experience and adherence to remote patient management in chronic heart failure patients: a posthoc analysis of the TIM-HF2 trial | 2023 | European Journal of Cardiovascular Nursing | 22 | 245-253 |
| 272 | Prichard, R. A. et al. | Discrepancies between proxy estimates and patient reported, health related, quality of life: minding the gap between patient and clinician perceptions in heart failure | 2021 | Quality of Life Research | 30 | 1049-1059 |
| 273 | Purcell, C. et al. | Home-based cardiac rehabilitation for people with heart failure and their caregivers: a mixed-methods analysis of the roll out an evidence-based programme in Scotland (SCOT:REACH-HF study) | 2023 | European Journal of Cardiovascular Nursing | 22 | 804-813 |
| 274 | Qian, F. et al. | Racial Differences in Heart Failure Outcomes: Evidence From Telemonitoring to Improve Heart Failure Outcomes (Tele-HF) Trial | 2015 | JACC Heart Failure | 3(7) | 531-538 |
| 275 | Quan, H. et al. | Opinions on chelation therapy in patients undergoing coronary angiography: Cross-sectional survey | 2007 | The Canadian Journal of Cardiology | 23(8) | 635-640 |
| 276 | Quartieri, F. et al. | New insertable cardiac monitors show high diagnostic yield and good safety profile in real-world clinical practice: results from the international prospective observational SMART Registry | 2023 | Europace | 25 | 1-10 |
| 277 | Rasmussen, A. A. et al. | Predictors of patient-reported outcomes at discharge in patients with heart failure | 2020 | European Journal of Cardiovascular Nursing | 19(8) | 748-756 |
| 278 | Rasmussen, A. A. et al. | Prognostic impact of self-reported health on clinical outcomes in patients with heart failure | 2021 | European Heart Journal – Quality of Care and Clinical Outcomes | 7 | 397-406 |
| 279 | Rasmussen, A. A. et al. | Patient-reported outcomes and medication adherence in patients with heart failure | 2021 | European Heart Journal – Cardiovascular Pharmacotherapy | 7 | 287-295 |
| 280 | Rasmussen, A. A. et al. | Gender differences in patient-reported outcomes in patients with acute myocardial infarction | 2022 | European Journal of Cardiovascular Nursing | 21 | 772-781 |
| 281 | Rasmussen, T. B. et al. | Subgroup Differences and Determinants of Patient-Reported Mental and Physical Health in Patients With Ischemic Heart Disease | 2019 | Journal of Cardiovascular Nursing | 34(4) | E11-E21 |
| 282 | Rasmussen, T. B. et al. | Patient-reported outcomes, sociodemographic and clinical factors are associated with 1-year mortality in patients with ischemic heart disease—fndings from the DenHeart cohort study | 2022 | Quality of Life Research | 31 | 389-402 |
| 283 | Rawal, S. et al. | Impact of Intravascular Brachytherapy on Patient-Reported Outcomes in Patients with Coronary Artery Disease | 2020 | Cardiovascular Revascularization Medicine | 21 | 1550-1554 |
| 284 | Reading Turchioe, M. et al. | Older Adults Can Successfully Monitor Symptoms Using an Inclusively designed mobile application | 2020 | Journal of the American Geriatrics Society | 68(6) | 1313-1318 |
| 285 | Reading Turchioe, M. et al. | Visual analogies, not graphs, increase patients' comprehension of changes in their health status | 2020 | J Am Med Inform Assoc | 27(5) | 677-689 |
| 286 | Reddy, Y. N. V. et al. | Quality of life in heart failure with preserved ejection fraction: importance of obesity, functional capacity, and physical inactivity | 2020 | European Journal of Heart Failure | 22 | 1009-1018 |
| 287 | Reif, S. et al. | Supporting patients with heart failure with digital therapeutics—A pilot study in Germany | 2022 | Digital Health | 8 | 1-8 |
| 288 | Reilly, C. M. et al. | Single Subject Design: Use of Time Series Analyses in a Small Cohort to Understand Adherence with a Prescribed Fluid Restriction | 2015 | Applied nursing research: ANR | 28(4) | 356-365 |
| 289 | Richards, B. G. et al. | Patient-Reported Quality of Hospital Discharge Transitions: Results from the SILVER-AMI Study | 2020 | Journal of general internal medicine | 35(3) | 808-814 |
| 290 | Riley, D. L. et al. | Continuity of Cardiac Care: Cardiac Rehabilitation Participation and Other Correlates | 2007 | International Journal of Cardiology | 119(3) | 326-333 |
| 291 | Risom, S. S. et al. | High Readmission Rates and Mental Distress 1 yr After Ablation for Atrial Fibrillation or Atrial Flutter | 2019 | Journal of cardiopulmonary rehabilitation and prevention | 39(1) | 33-38 |
| 292 | Risom, S. S. et al. | Cardiac Rehabilitation for Patients Treated for Atrial Fibrillation With Ablation Has Long-Term Effects 12-and 24-Month Follow-up Results From the Randomized CopenHeartRFA Trial | 2020 | Archives of Physical Medicine and Rehabilitation | 101(11) | 1877-1886 |
| 293 | Risom, S. S. et al. | Association Between Risk Factors and Readmission for Patients With Atrial Fibrillation Treated With Catheter Ablation: Results From the Nationwide DenHeart Study | 2023 | Journal of Cardiovascular Nursing | 38(1) | E31-E39 |
| 294 | Roch, C. et al. | Utility of the integrated palliative care outcome scale (IPOS): a cross-sectional study in hospitalised patients with heart failure | 2020 | European Journal of Cardiovascular Nursing | 19(8) | 702-710 |
| 295 | Roikjær, S. G. et al. | Qualitative study to explore what patients with heart failure find significant during integrated palliative care sessions in a Danish clinic | 2020 | BMJ Open | 10 | e043955 |
| 296 | Rollman, B. L. et al. | Efficacy of Blended Collaborative Care for Patients With Heart Failure and Comorbid Depression: A Randomized Clinical Trial | 2021 | JAMA Internal Medicine | 181(10) | 1369-1380 |
| 297 | Rotvig, C. et al. | Unreadiness for hospital discharge predicts readmission among cardiac patients: results from the national DenHeart survey | 2021 | European Journal of Cardiovascular Nursing | 20 | 667-675 |
| 298 | Rowin, E. J. et al. | Assessing the impact of atrial fibrillation on symptoms and quality of life in hypertrophic cardiomyopathy | 2024 | Journal of cardiovascular electrophysiology | 35(4) | 654-663 |
| 299 | Rubio, R. et al. | Quality of life and disease experience in patients with heart failure with reduced ejection fraction in Spain: a mixed-methods study | 2021 | BMJ Open | 11 | e053216 |
| 300 | Sadlonova, M. et al. | Generalized anxiety is a predictor of impaired quality of life in patients with atrial fibrillation: Findings from the prospective observational ARENA study | 2024 | Journal of Psychosomatic Research | 176 | 111542 |
| 301 | Salisbury, A. C. et al. | Outcomes of Medical Therapy Plus PCI for Multivessel or Left Main CAD Ineligible for Surgery | 2023 | JACC: Cardiovascular Interventions | 16(3) | 261-273 |
| 302 | Sampson, F. C. et al. | Is primary angioplasty an acceptable alternative to thrombolysis? Quantitative and qualitative study of patient and carer satisfaction | 2010 | Health Expect | 13(4) | 350-358 |
| 303 | Sandhu, A. T. et al. | Impact of Patient-Reported Outcome Measurement in Heart Failure Clinic on Clinician Health Status Assessment and Patient Experience: A Sub-Study of the PRO-HF Trial | 2023 | Circulation. Heart Failure | 16(2) | e010280 |
| 304 | Sandhu, U. et al. | Patient-Reported Outcomes in a Multidisciplinary Electrophysiology-Psychology Ventricular Arrythmia Clinic | 2022 | Journal of the American Heart Association | 11 | e025301 |
| 305 | Sapontis, J. et al. | Early Procedural and Health Status Outcomes After Chronic Total Occlusion Angioplasty: A Report From the OPEN-CTO Registry (Outcomes, Patient Health Status, and Efficiency in Chronic Total Occlusion Hybrid Procedures) | 2017 | JACC: Cardiovascular Interventions | 10(15) | 1523-1534 |
| 306 | Sato, T. et al. | Long-term preservation of functional capacity and quality of life in advanced heart failure patients with bridge to transplant therapy: A report from Japanese nationwide multicenter registry | 2022 | International Journal of Cardiology | 356 | 66-72 |
| 307 | Schluep, M. et al. | In-depth assessment of health-related quality of life after in-hospital cardiac arrest | 2022 | Journal of Critical Care | 68 | 22-30 |
| 308 | Schmitz, B. et al. | Living Lab Data of Patient Needs and Expectations for eHealth-Based Cardiac Rehabilitation in Germany and Spain From the TIMELY Study: Cross-Sectional Analysis | 2024 | Journal of Medical Internet Research | 26 | e53991 |
| 309 | Schofield, T. et al. | Patient experiences using a novel tool to improve care transitions in patients with heart failure: a qualitative analysis | 2019 | BMJ Open | 9 | e026822 |
| 310 | Scordo, K. A. | Mitral valve prolapse syndrome health concerns, symptoms, and treatments | 2005 | Western Journal of Nursing Research | 27(4) | 390-405 |
| 311 | Sears, S. F. et al. | Defibrillator shocks and their effect on objective and subjective patient outcomes: results from the painfree SST clinical trial | 2018 | Heart Rhythm | 15(5) | 734-740 |
| 312 | Seki, Y. et al. | Catheter ablation improves outcomes and quality of life in Japanese patients with early-stage atrial fibrillation: A retrospective cohort study | 2022 | Heart Rhythm | 19 | 1076-1083 |
| 313 | Seo, M. et al. | The clinical relevance of quality of life in heart failure patients with preserved ejection fraction | 2023 | ESC Heart Failure | 10 | 995-1002 |
| 314 | Shang, P. et al. | Association Between Medication Adherence and 1-Year Major Cardiovascular Adverse Events After Acute Myocardial Infarction in China | 2019 | Journal of the American Heart Association | 8(9) | e011793 |
| 315 | Shoji, S. et al. | Discrepancy between patient-reported quality of life and the prognostic assessment of Japanese patients hospitalized with acute heart failure | 2019 | Heart and Vessels | 34 | 1464-1470 |
| 316 | Shufelt, C. L. et al. | Biometric and Psychometric Remote Monitoring and Cardiovascular Risk Biomarkers in Ischemic Heart Disease | 2020 | Journal of the American Heart Association | 9 | e016023 |
| 317 | Siminoff, L. A. et al. | Barriers to obtaining diagnostic testing for coronary artery diagnostic testing for coronary artery disease among veterans | 2008 | American Journal of Public Health | 98(12) | 2207-2213 |
| 318 | Six, S. et al. | Patient-reported outcome measures on mental health and psychosocial factors in patients with Brugada syndrome | 2023 | Europace | 25 | 1-10 |
| 319 | Sohn, A. et al. | Integrating remote monitoring into heart failure patients' care regimen: A pilot study | 2020 | PloS one | 15(11) | e0242210 |
| 320 | Speier, W. et al. | Evaluating utility and compliance in a patient-based eHealth study using continuous-time heart rate and activity trackers | 2018 | Journal of the American Medical Informatics Association | 25(10) | 1386-1391 |
| 321 | Spertus, J. V. et al. | Integrating Quality of Life and Survival Outcomes in Cardiovascular Clinical Trial: Results from the PARTNER Trial | 2019 | Circulation. Cardiovascular quality and outcomes | 12(6) | e005420 |
| 322 | Stein, G. et al. | Ethnic differences in quality of life and its association with survival in patients with heart failure | 2020 | Clinical Cardiology | 43(9) | 976-985 |
| 323 | Steinberg, B. A. et al. | Factors Associated With Large Improvements in Health-Related Quality of Life in Patients with Atrial Fibrillation: Results from the Outcomes Registry for Better Informed Treatment of Atrial Fibrillation (ORBIT-AF) | 2020 | Circulation. Arrhythmia and electrophysiology | 13(5) | e007775 |
| 324 | Steinberg, B. A. et al. | Systematic collection of patient-reported outcomes in atrial fibrillation: feasibility and initial results of the Utah mEVAL AF programme | 2020 | Europace | 22 | 369-374 |
| 325 | Steinberg, B. A. et al. | Patient-reported outcomes and costs associated with vascular closure and Same-Day Discharge following Atrial Fibrillation Ablation | 2022 | Journal of cardiovascular electrophysiology | 33(8) | 1737-1744 |
| 326 | Steinberg, B. A. et al. | Quantifying the Impact of Atrial Fibrillation on HF-Related Patient-Reported Outcomes in the Utah mEVAL Program | 2022 | Journal of cardiac failure | 28(1) | 13-20 |
| 327 | Steinberg, L. et al. | HeartFull: Feasibility of an Integrated Program of Care for Patients with Advanced Stage of Heart Failure | 2022 | American Journal of Hospice & Palliative Medicine | 39(10) | 1194-1202 |
| 328 | Stewart, G. C. et al. | Ambulatory Advanced Heart Failure in Women: A Report From the REVIVAL Registry | 2019 | JACC: Heart Failure | 7(7) | 602-611 |
| 329 | Strangl, F. et al. | Symptom burden, psychosocial distress and palliative care needs in heart failure-A cross-sectional explorative pilot study | 2023 | Clinical Research in Cardiology | 112 | 49-58 |
| 330 | Styra, R. et al. | Toronto aortic stenosis quality of life questionnaire (TASQ): validation in TAVI patients | 2020 | BMC Cardiovascular Disorders | 20 | 209 |
| 331 | Tabaja, C. et al. | Impact of obesity on catheter ablation of atrial fibrillation: Patients characteristics, procedural complications, outcomes, and quality of life | 2023 | Journal of cardiovascular electrophysiology | 34(8) | 1648-1657 |
| 332 | Tang, L. et al. | Transcatheter aortic valve replacement in patients with severe comorbidities: A retrospective cohort study | 2021 | Catheter Cardiovasc Interv | 97(2) | E253-E262 |
| 333 | Taylor, C. J. et al. | From breathless to failure: symptom onset and diagnostic meaning in patients with heart failure —  a qualitative study | 2017 | BMJ Open | 7 | e013648 |
| 334 | Teramoto, K. et al. | Patient-Reported Versus Physician-Assessed Health Status in Heart Failure With Reduced and Preserved Ejection Fraction From ASIAN-HF Registry | 2023 | Circulation: Cardiovascular Quality and Outcomes | 16 | e009134 |
| 335 | Theunissen, Ljhj et al. | The prognostic value of quality of life in atrial fibrillation on patient value | 2023 | Health and quality of life outcomes | 21(1) | 33 |
| 336 | Thompson, J. H. et al. | Adverse events in patients with a left ventricular assist device: are patient-reported outcomes affected? | 2022 | European Journal of Cardiovascular Nursing | 21 | 254-260 |
| 337 | Thorup, C. B. et al. | Are patient-reported outcomes at discharge associated with employment status after cardiac hospitalization? Results from the national DenHeart study | 2022 | European Journal of Cardiovascular Nursing | 21 | 453-463 |
| 338 | Thrysoee, L. et al. | Patient-reported outcomes at hospital discharge among patients arrhythmia: Results from the national DenHeart survey | 2020 | European Journal of Cardiovascular Nursing | 19(3) | 248-259 |
| 339 | Tian, J. et al. | CHF-PROM: validation of a patient-reported outcome measure for patients with chronic heart failure | 2018 | Health and Quality of Life Outcomes | 16 | 51 |
| 340 | Tian, J. et al. | Assessment of chronic disease self-management in patients with chronic heart failure based on the MCID of patient-reported outcomes by the multilevel model | 2021 | BMC Cardiovascular Disorders | 21 | 58 |
| 341 | Tian, J. et al. | Machine learning prognosis model based on patient-reported outcomes for chronic heart failure patients after discharge | 2023 | Health and Quality of Life Outcomes | 21 | 31 |
| 342 | Timóteo, A. T. et al. | Quality of life in adults living in the community with previous self-reported myocardial infarction | 2020 | Revista Portuguesa de Cardiologia | 39(7) | 367-373 |
| 343 | Tjustrup, N. C. et al. | Patient-reported outcomes, clinical, and demographic variables as predictors of withdrawal from the workforce after hospitalization with heart failure: finding from the national DenHeart survey | 2022 | European Journal of Cardiovascular Nursing | 21 | 332-340 |
| 344 | Toledo-Chávarri, A. et al. | Co-Design Process of a Virtual Community of Practice for the Empowerment of People with Ischemic Heart Disease | 2020 | International journal of integrated care | 20(4) | 9 |
| 345 | Tower-Rader, A. et al. | Patient reported outcomes in obstructive hypertrophic cardiomyopathy undergoing myectomy: Results from SPIRIT-HCM study | 2023 | Progress in Cardiovascular Diseases | 80 | 66-73 |
| 346 | Trevino, K. M. & McConnell, T. R. | Religiosity and Spirituality During Cardiac Rehabilitation: A Longitudinal Evaluation of Patient-Reported Outcomes and Exercise Capacity | 2015 | Journal of cardiopulmonary rehabilitation and prevention | 35(4) | 246-254 |
| 347 | Trivedi, R. et al. | Angina symptom burden associated with depression status among veterans with Ischemic heart disease | 2015 | Ann Behav Med | 49(1) | 58-65 |
| 348 | Vaanholt, M. C. W. et al. | Perceived advantages and disadvantages of oral anticoagulants, and the trade-offs patients make in choosing anticoagulant therapy and adhering to their drug regimen | 2018 | Patient Education and Counseling | 101 | 1982-1989 |
| 349 | Van Schalkwijk, D. L. et al. | ShareHeart: A patient journey map of patients with ischemia and non-obstructive coronary artery disease based on qualitative research | 2023 | Journal of Clinical Nursing | 32(13-14) | 3434-3444 |
| 350 | Verdu-Rotellar, J. M. et al. | Impact of malnutrition on the quality of life in older patients with advanced heart failure: a cohort study | 2024 | Revista Clínica  Española | 224 | 105-113 |
| 351 | Versteeg, H. et al. | Effect of remote monitoring on patient-reported outcomes in European heart failure patients with an impantable cardioverter-defibrillator: primary results of the REMOTE-CIED randomized trial | 2019 | Europace | 21 | 1360-1368 |
| 352 | Viktorisson, A. et al. | Well-being among survivors of out-of-hospital cardiac arrest: a cross-sectional retrospective study in Sweden | 2018 | BMJ Open | 8 | e021729 |
| 353 | Vyshnevska, I. R. et al. | THE ROLE OF BIOCHEMICAL MARKERS AND PATIENT-REPORTED OUTCOMES IN PREDICTING COMPOSITE ONE-YEAR ENDPOINT IN ST-SEGMENT ELEVATION MYOCARDIAL INFARCTION | 2023 | Polski  Merkuriusz  Lekarski | 51(1) | 21-29 |
| 354 | Vámosi, M. et al. | Patient-reported outcomes predict high readmission rates among patients with cardiac diagnoses. Findings from the DenHeart study | 2020 | International Journal of Cardiology | 300 | 268-275 |
| 355 | Wagner, M. K. et al. | Sex Differences in Patient-Reported Outcomes in the Immediate Recovery Period After Resuscitation: Findings From the Cross-sectional DenHeart Survey | 2023 | Journal of Cardiovascular Nursing | 38(3) | 279-287 |
| 356 | Walfridsson, U. et al. | Development and validation of a new Arrhythmia-Specific questionnaire in Tachycardia and Arrythmia (ASTA) with focus on symptom burden | 2012 | Health and Quality of Life Outcomes | 10 | 44 |
| 357 | Walfridsson, U. et al. | Gender and age differences in symptoms and health-related quality of life in patients with atrial fibrillation referred for catheter ablation | 2019 | Pacing and clinical electrophysiology: PACE | 42(11) | 1431-1439 |
| 358 | Walfridsson, U. et al. | Symptoms and health-related quality of life 5 years after catheter ablation of atrial fibrillation | 2022 | Clinical cardiology | 45(1) | 42-50 |
| 359 | Walsh, M. et al. | Heart failure symptom burden in outpatient cardiology: observational cohort study | 2024 | BMJ supportive & palliative care | 13(e3) | e1280–e1284 |
| 360 | Walthall, H. et al. | The development of a patient reported outcome measure for fatigue and breathlessness for patients with chronic heart failure (OxFAB) | 2023 | Heart & Lung | 58 | 116-124 |
| 361 | Wang, M. et al. | Trajectories of perceived social support in acute coronary syndrome | 2019 | Quality of Life Research | 28 | 1365-1376 |
| 362 | Wasserlauf, J. et al. | Patient-reported outcomes after cryoballoon ablation are equivalent between moderate sedation and general anaesthesia | 2020 | Journal of cardiovascular electrophysiology | 31(7) | 1579-1584 |
| 363 | Watson, D. E. et al. | Patient-reported experiences and outcomes following hospital care are associated with risk of readmission among adults with chronic health conditions | 2022 | PloS one | 17(11) | e0276812 |
| 364 | Werhahn, S. M. et al. | Designing meaningful outcome parameters using mobile technology: a new mobile application for telemonitoring of patients with heart failure | 2019 | ESC Heart Failure | 6 | 516-525 |
| 365 | White, J. et al. | Cardiff cardiac ablation patient-reported outcome measure (C-CAP): validation of a new questionnaire set for patients undergoing catheter ablation for cardiac arrythmias in the UK | 2016 | Qual Life Res | 25 | 1571-1583 |
| 366 | Wiandt, J. V. et al. | Patient reported symptom outcomes during medication titration for adult heart failure management | 2020 | Applied Nursing Research | 56 | 151342 |
| 367 | Withers, K. L. et al. | Patient reported outcome measures for cardiac ablation procedures: a multicentre pilot to develop a new questionnaire | 2014 | Europace | 16 | 1626-1633 |
| 368 | Wittboldt, S. et al. | Reliability of two questionnaires on physical function in patients with stable coronary artery disease | 2016 | European Journal of Cardiovascular Nursing | 15(2) | 142-149 |
| 369 | Wohlfahrt, P. et al. | Quality of Life in Patients With Heart Failure With Recovered Ejection Fraction | 2021 | Heart failure clinics | 16(4) | 409-420 |
| 370 | Wood, K. A. et al. | Development and initial psychometric evaluation of the Patient Perspective of Arrhythmia Questionnaire | 2009 | Research in nursing & health | 32(5) | 504-516 |
| 371 | Wood, K. A. et al. | Symptom challenges after atrial fibrillation ablation | 2017 | Heart & lung: the journal of critical care | 46(6) | 425-431 |
| 372 | Wu, C. J. et al. | Cardiac-diabetes self-management program for Australians and Taiwanese: A randomized blocked design study | 2017 | Nursing and Health Sciences | 19 | 307-315 |
| 373 | Wu, J. R. & Moser, D. K. | Health-Related Quality of Life Is a Mediator of the Relationship Between Medication Adherence and Cardiac Event-Free Survival in Patients with Heart Failure | 2021 | Journal of cardiac failure | 27(8) | 848-856 |
| 374 | Wyrwich, K. W. et al. | Triangulating Patient and Clinician Perspectives on Clinically Important Differences in Health-Related Quality of Life among Patients with Heart Disease | 2007 | Health services research | 42(6 Pt 1) | 2257-2323 |
| 375 | Xiong, G. L. et al. | Health Status and Depression Remission in Patients with Chronic Heart Failure: Patient-Reported Outcomes from the SADHART-CHF Trial | 2012 | Circulation. Heart failure | 5(6) | 688-692 |
| 376 | Yamada, S. et al. | Cell Therapy Improves Quality-of-Life in Heart Failure: Outcomes From a Phase III Clinical Trial | 2024 | Stem Cells Translational Medicine | 13 | 116-124 |
| 377 | Yang, J. X. et al. | Association between procedure appropriateness and patient-reported outcomes after percutaneous coronary intervention | 2020 | Heart (British Cardiac Society) | 106(6) | 441-446 |
| 378 | Yee, D. et al. | Comparison of the Kansas City Cardiomyopathy Questionnaire and Minnesota Living With Heart Failure Questionnaire in Predicting Heart Failure Outcomes | 2019 | The American journal of cardiology | 123(5) | 807-812 |
| 379 | Yu, D. S. et al. | Effectiveness and Cost-effectiveness of an Empowerment-Based Self-care Education Program on Health Outcomes Among Patients With Heart Failure: A Randomized Clinical Trial | 2022 | JAMA network open | 5(4) | e225982 |
| 380 | Zenger, B. et al. | Patient Reported Outcomes and Subsequent Management in Atrial Fibrillation Clinical Practice: Results from the Utah mEVAL AF Program | 2020 | Journal of cardiovascular electrophysiology | 31(12) | 3187-3195 |
| 381 | Zhang, X. et al. | Prognostic value of patient-reported outcomes in predicting 30 day all-cause readmission among older patients with heart failure | 2022 | ESC Heart Failure | 9 | 2840-2850 |
| 382 | Zhu, C. et al. | Impact of Marital Stress on 1-Year Health Outcomes Among Young Adults With Acute Myocardial Infarction | 2023 | Journal of the American Heart Association | 12 | e030031 |
| 383 | Zinckernagel, L. et al. | How to measure experiences of healthcare quality in Denmark among patients with heart disease? The development and psychometric evaluation of a patient-reported instrument | 2017 | BMJ Open | 7 | e016234 |
| 384 | Zinckernagel, L. et al. | Association of patient-reported psychosocial healthcare and risk of readmissions and mortality in patients with ischemic heart disease: A population-based cohort study | 2022 | Journal of Psychosomatic Research | 156 | 110776 |
| 385 | Zisiopoulou, M. et al. | A Novel Method to Predict Mortality and Length of Stay after Transfemoral Transcatheter Aortic Valve Implantation | 2021 | Medicina (Kaunas, Lithuania) | 57(12) | 1332 |
| 386 | van Kessel, P. et al. | Measuring patient outcomes in chronic heart failure: psychometric properties of the Care-Related Quality of Life survey for Chronic Heart Failure (CaReQoL CHF) | 2017 | BMC Health Services Research | 17 | 536 |
| 387 | van Montfort, E. et al. | Person-centered analysis of psychological traits to explain heterogeneity in patient-reported outcomes of coronary artery disease– the THORESCI study | 2018 | Journal of Affective Disorders | 236 | 14-22 |
| 388 | van Veen, B. et al. | Patient-Reported Quality of Life as a Predictor of  Mortality and Ventricular Tachyarrhythmia’s During  7 Years’ Follow-Up in Patients With an Implantable  Cardioverter Defibrillator (from the MIDAS Study) | 2019 | The American journal of cardiology | 123(4) | 605-610 |
| 389 | van Veghel, D. et al. | Improving clinical outcomes and patient satisfaction among patients with coronary artery disease: an example of enhancing regional integration between a cardiac centre and a referring hospital | 2020 | BMC Health Service Research | 20 | 494 |
| 390 | Ängerud, K. H. et al. | Areas for quality improvements in heart failure care: quality of care from the patient’s perspective | 2017 | Scandinavian journal of caring sciences | 31(4) | 830-838 |
