# Supplementary material for: Patient-reported outcome and experience measures in cardiovascular disease: a scoping review as part of iCARE4CVD
Source: J Patient Rep Outcomes. 2025 Dec 9;9:141. doi: 10.1186/s41687-025-00980-4 (PMC12696235; doi:10.1186/s41687-025-00980-4)
Supplement: Supplementary file 3 — Supplementary Material 3 [file 41687_2025_980_MOESM3_ESM.docx]

**Appendix 3: List of identified patient-reported outcome measures**

| Nr. | Measurement Instrument | Abbreviation | Type |
| --- | --- | --- | --- |
| 1 | 10-item Depression in Medical Illness | DMI-10 | Generic |
| 2 | 15-Dimensional health-related quality of life measure | 15D | Generic |
| 3 | 3-level EuroQOL 5 dimensions | EQ-5D-3L | Generic |
| 4 | 5-level EuroQOL 5 dimensions | EQ-5D-5L | Generic |
| 5 | Acceptance and Action Questionnaire – version 2 | AAQ-2 | Generic |
| 6 | Activities-specific Balance confidence scale | ABC-D | Generic |
| 7 | Arrhythmia-specific questionnaire in tachycardia and arrhythmia-HRQoL scale | ASTA HRQoL scale | Disease-specific |
| 8 | Arrhythmia-specific questionnaire in tachycardia and arrhythmia-symptom scale | ASTA symptom scale | Disease-specific |
| 9 | Atrial Fibrillation Effect on Quality-of-Life | AFEQT | Disease-specific |
| 10 | Atrial Fibrillation Impact Questionnaire | AFImpact | Disease-specific |
| 11 | Atrial Fibrillation Severity Scale | AFSS | Disease-specific |
| 12 | Atrial Fibrillation Symptom Severity Scale | AFSS | Disease-specific |
| 13 | Atrial Fibrillation-6 | AF6 | Disease-specific |
| 14 | Beck Anxiety Inventory | BAI | Disease-specific |
| 15 | Beck Depression Inventory - Second Edition | BDI-II | Disease-specific |
| 16 | Borg Rating of Perceived Exertion | Borg RPE | Generic |
| 17 | Brief Coping Orientation to Problems Experienced Inventory | Brief-COPE | Generic |
| 18 | Brief Illness Perception Questionnaire | B-IPQ | Generic |
| 19 | Brief Pain Inventory | BPI | Generic |
| 20 | Brief Resilient Coping Scale | BRCS | Generic |
| 21 | Canadian Classification Society Angina Grading System | CCS Angina Grading System | Disease-specific |
| 22 | Cantril’s Ladder of Life | Cantril Ladder | Generic |
| 23 | Cardiac Anxiety Questionnaire | CAQ | Disease-specific |
| 24 | Cardiac Denial of Impact Scale | CDIS | Disease-specific |
| 25 | Cardiac Health Profile | CHP | Disease-specific |
| 26 | Cardiff Cardiac Ablation PROM Tools (Cardiff Cardiac Ablation PROM - Pre-ablation, Cardiff Cardiac Ablation PROM - Post-ablation) | C-CAP1, C-CAP2 | Disease-specific |
| 27 | Care-Related Quality of Life survey for Chronic Heart Failure | CaReQoL CHF | Disease-specific |
| 28 | Center for Epidemiologic Studies Depression Scale | CES-D | Generic |
| 29 | Chronic Heart Failure - PRO | CHF-PROM | Disease-specific |
| 30 | Chronic heart failure health-related quality of life questionnaire | CHFQOLQ-20 | Disease-specific |
| 31 | Chronic Heart Failure Questionnaire | CHQ, CHFQ | Disease-specific |
| 32 | Chronic Heart Failure Questionnaire Self-Administered Standardized Format | CHQ-SAS | Disease-specific |
| 33 | Clinical Frailty Scale | CFS | Generic |
| 34 | Coping Inventory of Stressful Situations | CISS | Generic |
| 35 | Diabetes Management Self-Efficacy Scale | DMSES | Disease-specific |
| 36 | Diet History Questionnaire | DHQ | Generic |
| 37 | Dietary Instrument for Nutrition Education | DINE | Generic |
| 38 | Disability Rating Index | DRI | Generic |
| 39 | Dispositional Resilience Scale | DRS-15 | Generic |
| 40 | Distress Thermometer | DT | Generic |
| 41 | Duke Activity Status Index | DASI | Generic |
| 42 | Eating Habits Confidence Survey | EC | Generic |
| 43 | Edmonton Symptom Assessment System | ESAS | Generic |
| 44 | Epworth Sleepiness Scale | ESS | Generic |
| 45 | European Heart Rhythm Association score of atrial fibrillation | EHRA score | Disease-specific |
| 46 | EuroQOL 5 dimensions | EQ-5D | Generic |
| 47 | EuroQOL visual analogue scale | EQ-VAS | Generic |
| 48 | Exercise Vital Sign | EVS | Generic |
| 49 | Eysenck Personality Questionnaire | EPQ | Generic |
| 50 | Fatigue Assessment Scale | FAS | Disease-specific |
| 51 | Fatigue Impact Scale | FIS | Disease-specific |
| 52 | Fatigue Severity Scale | FSS | Disease-specific |
| 53 | Food Frequency Questionnaire | FFQ | Generic |
| 54 | Fried Frailty Phenotype | FFP | Generic |
| 55 | Frontal Assessment Battery | FAB | Disease-specific |
| 56 | Functional Assessment of Cancer Therapy - General | FACT-G | Disease-specific |
| 57 | Functional Assessment of Chronic Illness Therapy - Fatigue | FACIT-F | Generic |
| 58 | Functional Assessment of Chronic Illness Therapy - Palliative Care | FACIT-Pal | Generic |
| 59 | Functional Assessment of Chronic Illness Therapy - Spiritual Well-Being | FACIT-Sp-12 | Generic |
| 60 | General Health Questionnaire - 12 Items | GHQ-12 | Generic |
| 61 | General Health Questionnaire - 60 Items | GHQ | Generic |
| 62 | General Symptom Distress Scale | GSDS | Generic |
| 63 | Generalized Anxiety Disorder - 2 | GAD-2 | Disease-specific |
| 64 | Generalized Anxiety Disorder - 7 | GAD-7 | Disease-specific |
| 65 | Geriatric Depression Scale | GDS | Generic |
| 66 | Global Mood Scale | GMS | Generic |
| 67 | Godin and Shephard leisure–time physical activity questionnaire | GSLTPAQ | Generic |
| 68 | Hamilton Rating Scale for Depression | HRS-D | Disease-specific |
| 69 | Health Locus of Control Scale | HLC | Generic |
| 70 | Health Value Scale |  | Generic |
| 71 | Heart Failure Somatic Perception Scale | HFSPS | Disease-specific |
| 72 | Heart Failure Somatic Perception Scale - Dyspnea | HFSPS Dyspnea | Disease-specific |
| 73 | Heart Failure Symptom Scale | HFSS | Disease-specific |
| 74 | Heart Failure Symptom Survey | HFSS | Disease-specific |
| 75 | HeartQoL questionnaire | HeartQoL | Disease-specific |
| 76 | Hospital Anxiety and Depression Scale | HADS | Disease-specific |
| 77 | Hospital Anxiety and Depression Scale - Depressions | HADS-D | Disease-specific |
| 78 | Hyperarousal Behavioral Trait Scale | H-scale | Generic |
| 79 | Impact of Event Scale-Revised | IES-R | Disease-specific |
| 80 | Integrated Palliative Care Outcome Scale | IPOS | Generic |
| 81 | International Physical Activity Questionnaire | IPAQ | Generic |
| 82 | International Physical Activity Questionnaire - Short Form | IPAQ-SF | Generic |
| 83 | Kansas City Cardiomyopathy Questionnaire | KCCQ | Disease-specific |
| 84 | Kansas City Cardiomyopathy Questionnaire - 12 Items | KCCQ-12 | Disease-specific |
| 85 | Kansas City Cardiomyopathy Questionnaire Clinical Summary Score | KCCQ-CSS | Disease-specific |
| 86 | Kansas City Cardiomyopathy Questionnaire Physical Limitation Score | KCCQ-PLS | Disease-specific |
| 87 | Kansas City Cardiomyopathy Questionnaire Symptom Score | KCCQ-SS | Disease-specific |
| 88 | Kansas City Cardiomyopathy Questionnaire Total Symptom Score | KCCQ-TSS | Disease-specific |
| 89 | Karolinska Sleep Questionnaire sleep quality index | KSQ-sqi | Generic |
| 90 | MacNew Heart Disease Health-related Quality of Life Questionnaire | MacNew | Disease-specific |
| 91 | Medical Outcomes Study Sleep Scale | MOS-SS | Generic |
| 92 | Medical Research Council Dyspnoea Scale | MRC-DS | Disease-specific |
| 93 | Mini Nutritional Assessment Short Form | MNA-SF | Generic |
| 94 | Minimal Documentation System For Palliative Care | MIDOS | Generic |
| 95 | Minimal Insomnia Sleep Scale | MISS | Generic |
| 96 | Minnesota Living with Heart Failure Questionnaire | MLHFQ | Disease-specific |
| 97 | Modified Rankin Scale | mRS | Disease-specific |
| 98 | Montreal Cognitive Assessment | MoCA | Generic |
| 99 | Nottingham Health Profile | NHP | Generic |
| 100 | Outcomes and Assessment Information Set | OASIS | Generic |
| 101 | Oxford Fatigue And Breathlessness scale | OxFAB | Disease-specific |
| 102 | Patient Global Assessment Scale | PGA | Generic |
| 103 | Patient Global Impression of Change | PGI-C | Generic |
| 104 | Patient Global Impression of Severity | PGI-S | Generic |
| 105 | Patient Health Questionnaire - 2 Items | PHQ-2 | Disease-specific |
| 106 | Patient Health Questionnaire - 4 Items | PHQ-4 | Disease-specific |
| 107 | Patient Health Questionnaire - 8 Items | PHQ-8 | Disease-specific |
| 108 | Patient Health Questionnaire - 9 Items | PHQ-9 | Disease-specific |
| 109 | Patient Perception of Arrhythmia Questionnaire | PPAQ | Disease-specific |
| 110 | Patient Reported Outcomes Measurement Information System - 29 Profile | PROMIS-29 | Generic |
| 111 | Patient Reported Outcomes Measurement Information System - Global Health | PROMIS-Global Health | Generic |
| 112 | Patient-Reported Outcomes Measurement Information System - Anxiety | PROMIS-Anxiety | Disease-specific |
| 113 | Patient-Reported Outcomes Measurement Information System - Depression | PROMIS-Depression | Disease-specific |
| 114 | Patient-Reported Outcomes Measurement Information System - Fatigue | PROMIS-Fatigue | Generic |
| 115 | Patient-Reported Outcomes Measurement Information System - Pain Intensity | PROMIS-Pain Intensity | Generic |
| 116 | Patient-Reported Outcomes Measurement Information System - Pain Interference | PROMIS-Pain Interference | Generic |
| 117 | Patient-Reported Outcomes Measurement Information System - Physical Function | PROMIS-PF | Generic |
| 118 | Patient-Reported Outcomes Measurement Information System - Plus-Heart Failure | PROMIS-Plus-HF | Disease-specific |
| 119 | Patient-Reported Outcomes Measurement Information System - Plus-HF | PROMIS-Plus-HF | Disease-specific |
| 120 | Patient-Reported Outcomes Measurement Information System - Sexual Function | PROMIS-SexFS | Generic |
| 121 | Patient-Reported Outcomes Measurement Information System - Sleep Disturbance | PROMIS-Sleep Disturbance | Generic |
| 122 | Patient-Reported Outcomes Measurement Information System Short Form - Physical Function Samples with Mobility Aid Users | PROMIS Short Form – Physical Function Samples with Mobility Aid Users | Generic |
| 123 | Patient-Reported Outcomes Measurement Information System+Heart Failure-10 | PROMIS+HF-10 | Disease-specific |
| 124 | Patient-Reported Outcomes Measurement Information System+Heart Failure-27 | PROMIS+HF-27 | Disease-specific |
| 125 | Patient-Reported Outcomes Measurement Information System-Ability to Participate in Social Roles and Activities | PROMIS-Ability to Participate in Social Roles and Activities | Generic |
| 126 | Patient-Reported Outcomes Measurement Information System-Applied Cognition – Abilities | PROMIS-Applied Cognition – Abilities | Generic |
| 127 | Patient-Reported Outcomes Measurement Information System-Cognitive Function 8a | PROMIS-Cognitive Function | Generic |
| 128 | Patient-Reported Outcomes Measurement Information System-Dyspnea-Short Form | PROMIS-D-SF | Disease-specific |
| 129 | Patient-Reported Outcomes Measurement Information System-Fatigue-Short Form | PROMIS-F-SF | Disease-specific |
| 130 | Patient-Specific Functional Scale | PSFS | Generic |
| 131 | Perceived Health Competence Scale | PHCS | Generic |
| 132 | Perceived Stress Scale - 14 Items | PSS-14 | Generic |
| 133 | Perceived Stress Scale - 4 Items | PSS-4 | Generic |
| 134 | Physical Functioning-10 items scale of the SF-36 | PF-10 | Generic |
| 135 | Physical Health Component Scale of the SF-12 | PCS-12 | Generic |
| 136 | Piper Fatigue Scale Revised | PFS-R | Disease-specific |
| 137 | Pittsburgh Sleep Quality Index | PSQI | Generic |
| 138 | Positive and Negative Affect Schedule | PANAS | Generic |
| 139 | Powe Fatalism Inventory | PFI | Disease-specific |
| 140 | Profile of Mood States - Anxiety Subscale | POMS-A | Disease-specific |
| 141 | Profile of Mood States - Fatigue Subscale | POMS-F | Disease-specific |
| 142 | Psychological General Well-Being Index | PGWBI | Generic |
| 143 | Psychosocial Adjustment to Illness Scale | PAIS/PAIS-SR | Generic |
| 144 | PTSD Checklist - Civilian version | PCL-C | Disease-specific |
| 145 | Quality of Life at the End of Life | QUAL-E | Generic |
| 146 | Quality of Life Scale | QOLS | Generic |
| 147 | Quality of Recovery-40 | QoR-40 | Generic |
| 148 | Recent Physical Activity Questionnaire | RPAQ | Generic |
| 149 | Religiosity Measure | RM | Generic |
| 150 | Religious Coping Activities Scale | RCAS | Generic |
| 151 | Revised Life Orientation Test | LOT-R | Generic |
| 152 | Revised Urinary Incontinence Scale | RUIS | Generic |
| 153 | Rivermead Behavioral Memory Test | RBMT | Generic |
| 154 | Rose Dyspnea Scale | RDS | Disease-specific |
| 155 | Seattle Angina Questionnaire | SAQ | Disease-specific |
| 156 | Seattle Angina Questionnaire-7 | SAQ-7 | Disease-specific |
| 157 | Seattle Angina Questionnaire-Angina Frequency Subscale | SAQ-AF | Disease-specific |
| 158 | Seattle Angina Questionnaire-Physical Limitation Subscale | SAQ-PL | Disease-specific |
| 159 | Seattle Angina Questionnaire-Quality of Life Subscale | SAQ-QoL | Disease-specific |
| 160 | Self-Care Self-Efficacy Scale | SCSES | Generic |
| 161 | Self-Efficacy for Exercise | SEE | Generic |
| 162 | Self-Efficacy for Managing Chronic Disease | SEMCD | Generic |
| 163 | SF-12 Health Survey | SF-12 | Generic |
| 164 | SF-36 Health Survey | SF-36 | Generic |
| 165 | SF-36 Vitality Scale | SF-36 Vitality Scale | Generic |
| 166 | Sheehan Disability Scale | SDS | Generic |
| 167 | Social Interaction Anxiety Scale | SIAS | Generic |
| 168 | Starting the Conversation | STC | Generic |
| 169 | State Trait Anxiety Inventory | STAI | Disease-specific |
| 170 | Stockholms Marital Stress Scale | SMSS | Generic |
| 171 | Symbol Digit Modalities Test | SDMT | Disease-specific |
| 172 | Symptom Checklist: Frequency and Severity Scale | SCL | Disease-specific |
| 173 | Thirst Distress Scale | TDS-HF | Disease-specific |
| 174 | Toronto Aortic Stenosis Quality of Life Questionnaire | TASQ | Disease-specific |
| 175 | Type-D scale | DS14 | Generic |
| 176 | World Health Organization Quality of Life assessment instrument | WHOQOL-BREF | Generic |
