# Supplementary material for: Patient-reported outcome and experience measures in cardiovascular disease: a scoping review as part of iCARE4CVD
Source: J Patient Rep Outcomes. 2025 Dec 9;9:141. doi: 10.1186/s41687-025-00980-4 (PMC12696235; doi:10.1186/s41687-025-00980-4)
Supplement: Supplementary file 4 — Supplementary Material 4 [file 41687_2025_980_MOESM4_ESM.docx]

**Appendix 3:** **List of identified patient-reported experience measures**

| Nr. | Measurement Instrument | Abbreviation | Type |
| --- | --- | --- | --- |
| 1 | 5-item Decision Making Preference Questionnaire |  | Not validated |
| 2 | 9-item Shared Decision Making Questionnaire | SDM-Q-9 | Validated |
| 3 | Access to Knowledge Scale | AKS | Not validated |
| 4 | Adherence to Refills and Medications Scale | ARMS-7 | Validated |
| 5 | Adopted Prognosis and Treatment Perception Questionnaire | PTPQ | Not validated |
| 6 | Anti-Clot Treatment Scale | ACTS | Validated |
| 7 | Anticoagulation Knowledge Tool | AKT | Validated |
| 8 | Atlanta Heart Failure Knowledge Test | AHFKT | Validated |
| 9 | Atrial Fibrillation Knowledge Scale | AF-Knowledge Scale | Validated |
| 10 | Behavioral Risk Factor Surveillance System | BRFSS | Validated |
| 11 | Beliefs about Medicines Questionnaire | BMQ | Not validated |
| 12 | B-PREPARED Scale |  | Validated |
| 13 | Brief Health Literacy Screener | BHLS | Validated |
| 14 | Canadian Patient Experiences Survey - Inpatient Care | CPES-IC | Validated |
| 15 | Care Continuity Index | CCI | Validated |
| 16 | Care Transitions Measure | CTM-3 | Validated |
| 17 | Client Satisfaction Questionnaire | CSQ-8 | Validated |
| 18 | Duke-UNC Functional Social Support Questionnaire | FSSQ | Validated |
| 19 | Dutch Heart Failure Knowledge Scale | DHFKS | Validated |
| 20 | eHealth Literacy Scale | eHEALS | Validated |
| 21 | ENRICHD Social Support Instrument | ESSI | Validated |
| 22 | European Heart Failure Self-Care Behavior Scale | EHFScBS | Validated |
| 23 | Florida Patient Acceptance Scale | FPAS | Validated |
| 24 | Florida Shock Anxiety Scale | FSAS | Validated |
| 25 | Generic Short Patient Experiences Questionnaire | GS-PEQ | Not validated |
| 26 | Health Care Transition Measure | HCT | Validated |
| 27 | Health Information Technology Usability Evaluation Scale | Health-ITUES | Validated |
| 28 | Health Literacy Management Scale | HeLMS | Validated |
| 29 | Health Literacy Questionnaire | HLQ | Validated |
| 30 | Heart Continuity of Care questionnaire | HCCQ | Validated |
| 31 | Heart Failure Knowledge Scale | HFKS | Not validated |
| 32 | ICD Patient Concerns Questionnaire | ICDC | Validated |
| 33 | Illness Intrusiveness Rating Scale | IIRS | Validated |
| 34 | Jessa Atrial Fibrillation Knowledge Questionnaire | JAKQ | Validated |
| 35 | Knowledge Expectations of hospital patients Scale | KEhp-Scale | Not validated |
| 36 | Lubben Social Network Scale-6 | LSNS-6 | Validated |
| 37 | Medical Outcome Study Specific Adherence Scale | MOSAS | Validated |
| 38 | Medical Outcomes Study Social Support Scale | MOS-SS | Validated |
| 39 | Medication Adherence Rating Scale | MARS | Validated |
| 40 | Medication Knowledge Assessment Questionnaire | MKAQ | Validated |
| 41 | Miller Behavioral Style Scale | MBSS | Validated |
| 42 | Mishel Uncertainty Illness Scale | MUIS | Validated |
| 43 | Mitral Valve Prolapse Syndrome Standard Form | MVPS Standard Form | Not validated |
| 44 | modified Cancer Information Overload | modified-CIO | Not validated |
| 45 | Morisky Medication Adherence Scale | MMAS | Not validated |
| 46 | Multidimensional Scale of Perceived Social Support | MSPSS | Validated |
| 47 | Nordic Patient Experience Questionnaire | NORPEQ | Validated |
| 48 | Oslo Social Support Scale | OSSS-3 | Validated |
| 49 | Patient Activation Measure | PAM | Validated |
| 50 | Patient Satisfaction Scale | PSS | Not validated |
| 51 | Patient-Reported Outcomes Measurement Information System - Medication Adherence Scale | PMAS | Validated |
| 52 | Patient-Reported Outcomes Measurement Information System - Satisfaction with Participation in Discretionary Social Activities | PROMIS-Satisfaction with Participation in Discretionary Social Activities | Validated |
| 53 | Patient-Reported Outcomes Measurement Information System - Social Isolation | PROMIS-Social Isolation | Validated |
| 54 | Patient-Reported Outcomes Measurement Information System-Satisfaction with Social Roles and Activities | PROMIS-Satisfaction with Social Roles and Activities | Validated |
| 55 | Perception of Anticoagulant Treatment Questionnaire | PACT-Q | Validated |
| 56 | Quality from patients’ perspective Short Form | QPP | Validated |
| 57 | Rapid Estimate of Adult Literacy in Medicine | REALM-7 | Validated |
| 58 | Received Knowledge of hospital patients Scale | RKhp-Scale | Not validated |
| 59 | Response to Symptoms Questionnaire | RSQ | Not validated |
| 60 | Revised Heart Failure Compliance Questionnaire | RHFCQ | Validated |
| 61 | Self-Care of Heart Failure Index | SCHFI | Validated |
| 62 | Self-Care Questionnaire | SCQ | Not validated |
| 63 | Sense of Security in Care - Patients' Evaluation | SEC-P | Validated |
| 64 | Service Use Questionnaire - Social Services |  | Not validated |
| 65 | Short-form Test of Functional Health Literacy in Adults | S-TOFHLA | Validated |
| 66 | Social Support Rating Scale | SSRS | Validated |
| 67 | Summary of Diabetes Self-Care Activities | SDSCA | Validated |
| 68 | Survey of Healthcare Experiences of Patients | SHEP | Not validated |
| 69 | System Usability Scale | SUS | Validated |
| 70 | Treatment Burden Questionnaire | TBQ | Validated |
| 71 | Treatment Satisfaction Questionnaire for Medication | TSQM | Validated |
| 72 | Trust in Physician Scale | TIPS | Validated |
